# First observation of radiolytic bubble formation in unstirred nano-powder sludges and a consistent model thereof

**DOI:** 10.1038/s41598-021-01868-1

**Published:** 2021-11-24

**Authors:** Mel O’Leary, Aliaksandr Baidak, Martyn Barnes, Thomas Donoclift, Christopher Emerson, Catarina Figueira, Oliver Fox, Annette Kleppe, Aaron McCulloch, Darryl Messer, Robin Orr, Fred Currell

**Affiliations:** 1 Department of Chemistry, University of Manchester, Manchester, CA24 3HA UK; 2 Sellafield Ltd., Sellafield, CA20 1PG UK; 3 Scottish Universities Environmental Research Centre (SUERC), Glasgow, G75 0QF UK; 4 School of Maths and Physics, Queen’s University Belfast, Belfast, BT7 1NN UK; 5 Diamond Light Source Ltd., Didcot, OX11 0DE UK; 6 National Nuclear Lab, Warrington, WA3 6AE UK

**Keywords:** Energy transfer, Atomic and molecular interactions with photons, Surface chemistry, Nuclear waste, Soft materials, Heterogeneous catalysis, Electron transfer, Nanoparticles, Colloids

## Abstract

Experiments involving the irradiation of water contained within magnesium hydroxide and alumina nanoparticle sludges were conducted and culminated in observations of an increased yield of molecular hydrogen when compared to the yield from the irradiation of bulk water. We show that there is a relationship linking this increased yield to the direct nanoscale ionization mechanism in the nanoparticles, indicating that electron emission from the nanoparticles drives new radiative pathways in the water. Because the chemical changes in these sludges are introduced by irradiation only, we have a genuinely unstirred system. This feature allows us to determine the diffusivity of the dissolved gas. Using the measured gas production rate, we have developed a method for modelling when hydrogen bubble formation will occur within the nanoparticle sludges. This model facilitates the determination of a consistent radiolytic consumption rate coinciding with the observations of bubble formation. Thus, we demonstrate a nanoscale radiation effect directly influencing the formation of molecular hydrogen.

## Introduction

This paper investigates a heterogeneous chemical system where a nanopowder particulate phase is embedded inside a continuous aqueous phase. These systems are widespread in a range of disciplines, including healthcare^1–7^, and catalytic materials^8–12^. As such, we are especially concerned with heterogeneous chemical systems subject to ionizing radiation and the resultant radiolytic processes. One such process is the radiolytic hydrogen production in water due to radiation^13^. This production can be modified in mixtures of oxides or hydroxide nanoparticles with water. Additional reaction mechanisms are induced in these complex heterogeneous mixtures that do not occur in either the particulate or aqueous phase alone. The radiolytic hydrogen production in systems similar to those investigated in this paper, magnesium hydroxide (Mg(OH)$_{2}$) and alumina (Al$_{2}$O$_{3}$) sludges, is observed to be significantly enhanced compared to water alone^14–21^. This effect was initially reported by Petrik et al.^14^ with similar phenomena observed by LaVerne and co-workers on zirconia^15,16^, ceria^15^, urania^17^, alumina^18^, copper oxide^19^, ferrous oxide^20^ and bohemite^21^. The mechanisms for these increases in radiolytic product yields are varied and include exciton relaxation at or near zirconia surfaces^14^ and radiation driven oxidation of ferrous oxide surfaces^20^.

The heterogeneous chemical systems considered in this paper are directly related to nuclear waste handling, these systems are simple mimics for the waste materials formed from the corrosion of metal cladding on the nuclear fuel rods found in nuclear waste storage sites at Sellafield, UK, and Hanford, USA^22–27^. This model system can be used to elucidate key mechanisms which underpin the development of safe handling protocols for this waste. Heterogeneous radiation chemical systems with nanoparticles embedded in an aqueous phase are also a model system of the use of nanoparticle radio-enhancers to improve cancer radiotherapy^1^. Indeed, hafnium oxide nanoparticles are the first radiation nano-medicine to reach the market^2^. Heavy metal, especially gold, nanoparticles are also considered to be good candidates for improving radiotherapy^3,28,29^. These nanoparticles cluster around biologically sensitive targets in the cell where they locally increase energy deposition from irradiation^3–5,28^. They modify the radiation chemistry of water, by increasing the production of certain radiolytic products above the level expected from the increased energy deposition alone^1,4–7,28^.

**Figure 1:**
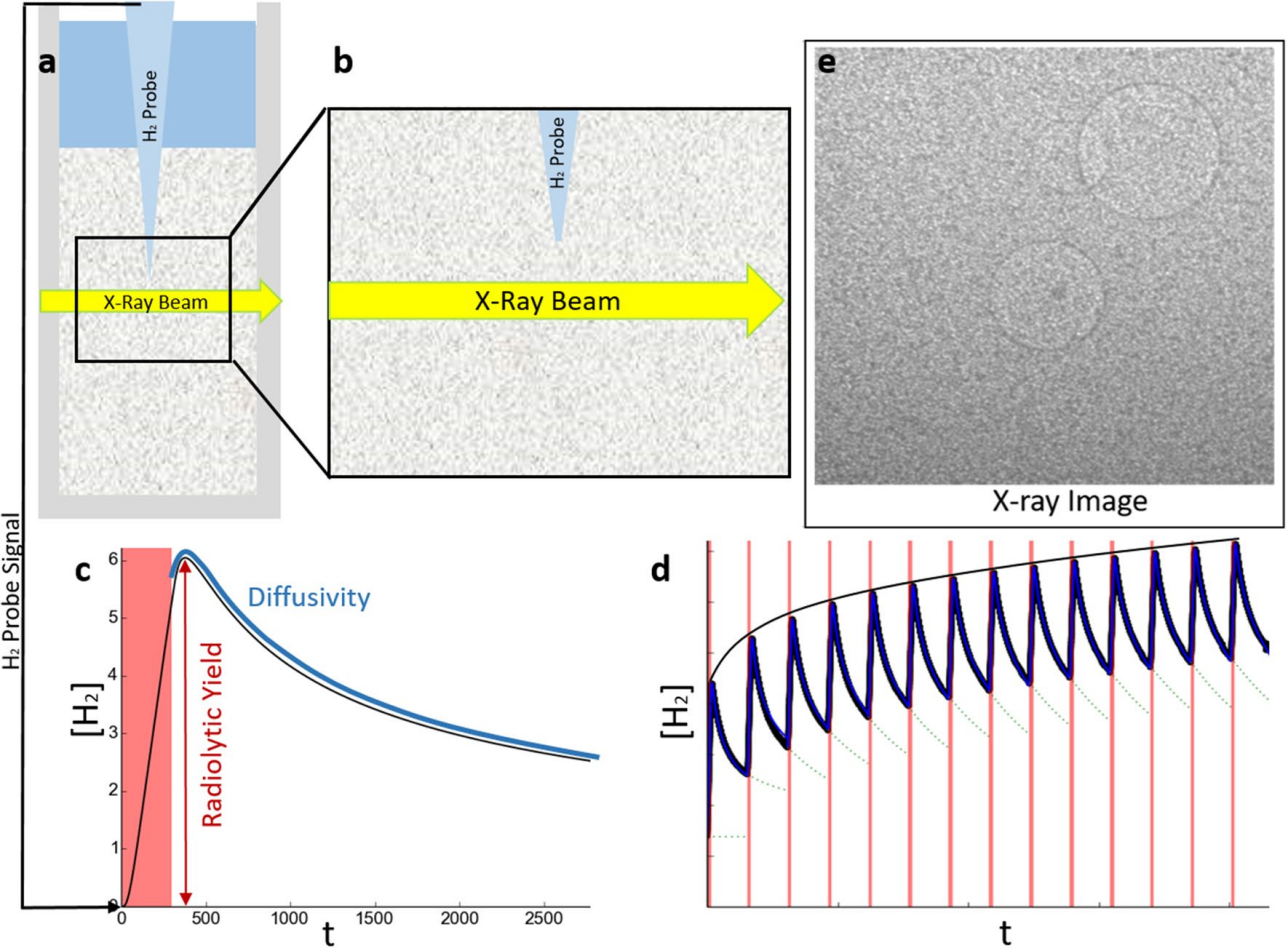
In a typical experiment, a cuvette filled with a nanoparticle sludge and hosting a hydrogen probe panel (**a**) was irradiated with a short and wide ribbon beam of monochromatic x-rays, approximately 1 mm below the tip of the probe, as illustrated in panel (**b**). The hydrogen probe signal was used to determine the radiolytic hydrogen yield and hydrogen diffusivity in the sludge, shown in panel (**c**: the red region indicates when the sample was irradiated. Over many irradiations, the hydrogen yield decreased to a lower rate, as depicted in panel (**d**) and indicated with the envelope curve. This decrease indicates radiolytic consumption, which we quantify in this paper. In a separate experiment, the sample was instead irradiated with an intense white beam from the B16 bending magnet source. A 2 mm $\times$ 2 mm x-ray image from the white beam irradiation, panel (**e**), shows the formation of bubbles. Figure made with plots plotted with matplotlib^55^ and an image processed by FIJI^56^.

This paper addresses the processes behind the increase in the radiolytic yield in water near magnesium hydroxide and alumina nanoparticles. However, what distinguishes this study from earlier work in the field is the combination of investigative research into both the product generation and its subsequent transport through the nanoscale heterogeneous system. Measurements of hydrogen concentration were made using hydrogen micro-sensors^30^ are described. These sensors allowed accurate measurement of the hydrogen concentration in a small volume. Radiolytic hydrogen generation was induced by the irradiation of the system with monochromatic x-rays at a number of discrete energies. The energy dependence of the radiolytic yield of hydrogen was used to deduce information about the hydrogen production mechanisms. A model for the radiolytic hydrogen production was then used to computationally model when radiolytic hydrogen bubbles would form. This model was used to determine the radiolytic consumption of the hydrogen by comparing the predicted and observed appearance time of bubbles. An overview of how the experiments were performed and how the model was formulated is illustrated in Fig. 1. The model developed here is universal and unrelated to the specific chemical production process. It applies to dissolved gas mass transport in most nano-particulate heterogeneous systems. This method of determining the diffusion coefficient can also be used to investigate tortuosity^31^ in heterogeneous phases.

**Figure 2:**
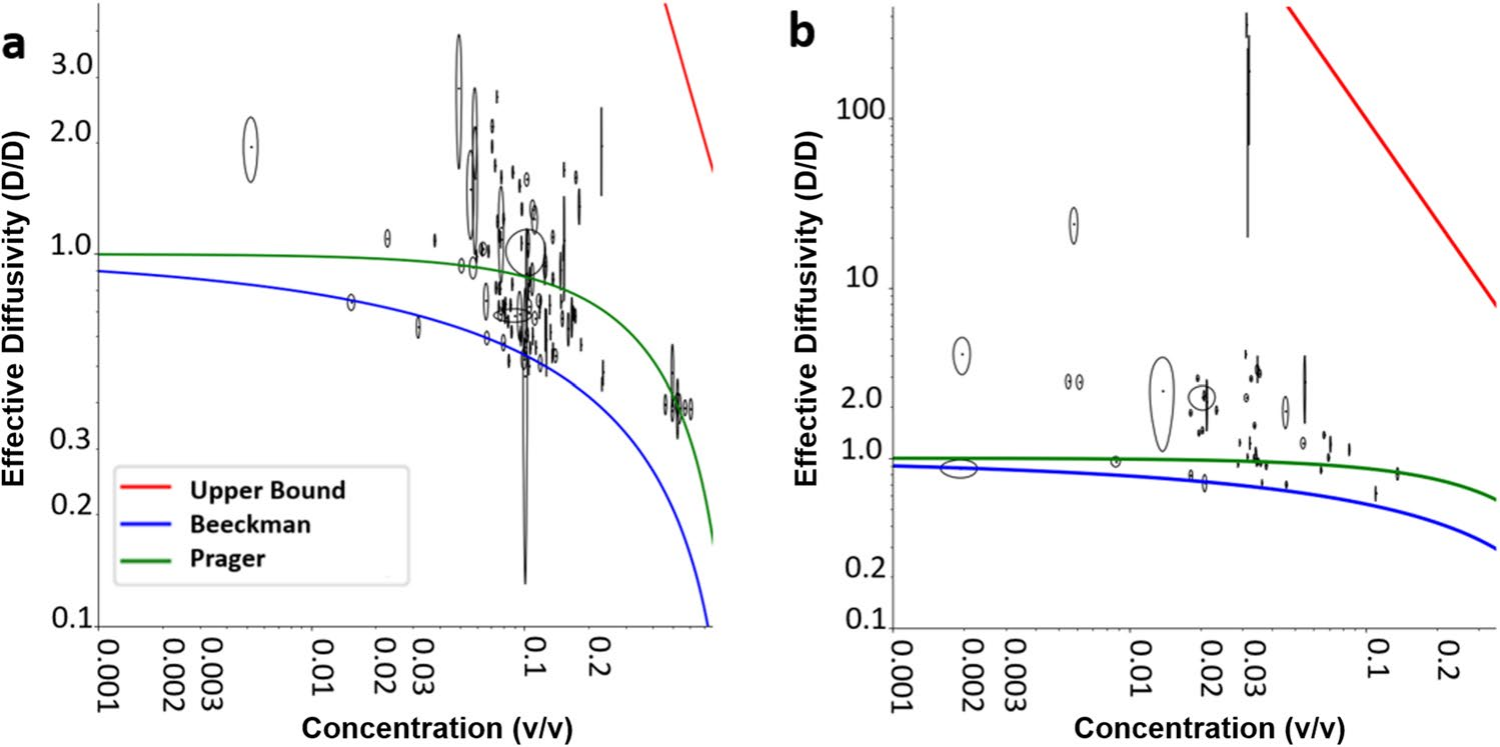
The measured hydrogen diffusivities compared to the two tortuosity models and the upper bound model (see text for details). Panel (**a**) shows the results for magnesium hydroxide. Panel (**b**) shows the results for alumina. The blue curves in panels (**a**) and (**b**) correspond to the Beeckman’s tortuosity model^39^. The green curve is the Prager upper limit model^40^. The red curves in panels (**a**) and (**b**) indicate the upper bound model, described by equation 1. Figure made with plots plotted with matplotlib^55^.

**Figure 3:**
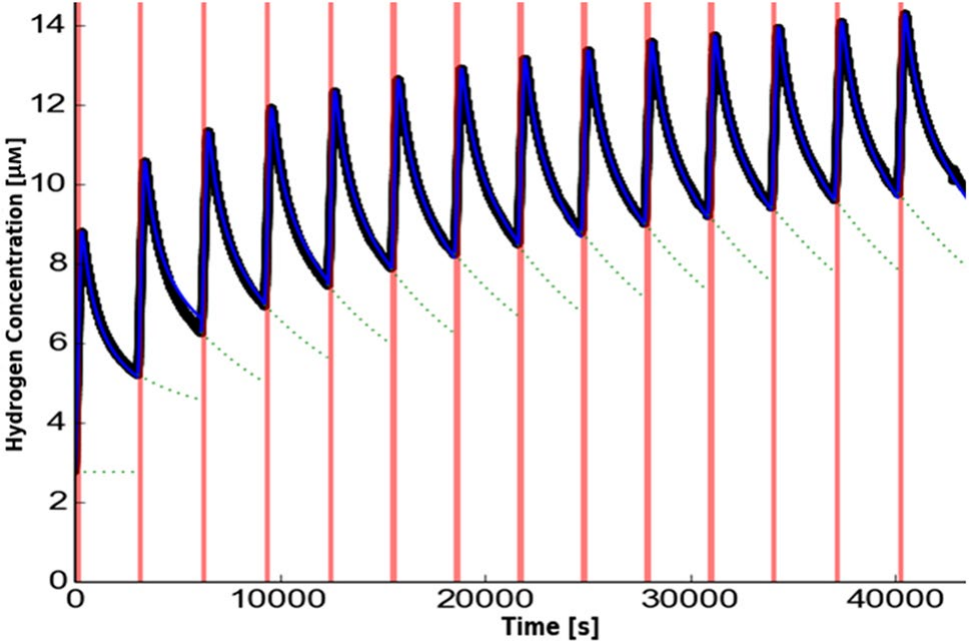
Hydrogen concentration as a function of time for one irradiation run and one cuvette. The black trace shows the measured data, the fit to a 1-dimensional diffusion model^33,49^ is shown in blue, the green dotted line shows the extrapolation of the background signal of hydrogen concentration from previous irradiations within the run. As the distance to the irradiated region and the start time are independently determined, there are two free parameters in the model: the diffusion coefficient, which determines the overall model shape, and the model parameter related to the radiolytic yield, which acts as an overall scaling factor. The results from these fits are reported in Table 1. Figure made with plot plotted with matplotlib^55^.

**Table 1:** The fitted results, and associated errors, for the runs plotted in Fig. 3. The covariance estimate between the fitted diffusivity and fitted radiolytic yield is also reported. The runs are in chronological order. The radiolytic yield determined from this data set was (0.107 ± 0.004) ${\upmu }$mol J$^{-1}$. The diffusivity calculated as a ratio of the hydrogen diffusion coefficient through the sludge to the hydrogen diffusion coefficient in water is 0.754 ± 0.014. The measured radiolytic yields indicate an observed radiolytic consumption of (27 ± 6) nmol J$^{-1}$. This gives a lower bound for the radiolytic consumption rate of (0.22 ± 0.08) nmolJ$^{-1}$ ${\upmu }$M$^{-1}$, given the change between the initial and second radiolytic yield, which would only hold if all the produced hydrogen remained in the irradiated region.

Run	Radiolytic yield (${\upmu }$molJ$^{-1}$)	Diffusivity (D/D)	Covariance estimate
1	0.1269 ± 0.0002	0.692 ± 0.005	0.0009
2	0.11610 ± 0.00016	0.792 ± 0.004	0.008
3	0.11068 ± 0.00016	0.762 ± 0.004	0.002
4	0.10892 ± 0.00011	0.779 ± 0.003	0.003
5	0.10779 ± 0.00012	0.773 ± 0.004	0.0017
6	0.10590 ± 0.00018	0.759 ± 0.006	0.0014
7	0.10293 ± 0.00013	0.754 ± 0.005	0.0017
8	0.10379 ± 0.00019	0.741 ± 0.004	0.0014
9	0.10091 ± 0.00013	0.747 ± 0.004	0.0013
10	0.10089 ± 0.00014	0.741 ± 0.003	0.0011
11	0.09971 ± 0.00011	0.751 ± 0.003	0.0011
12	0.09982 ± 0.00010	0.755 ± 0.003	0.0009
13	0.098996 ± 0.00010	0.737 ± 0.003	0.0014
14	0.099905 ± 0.00008	0.748 ± 0.002	0.003

## Results

The dissolved hydrogen concentration in irradiated sludges was measured over time for over an hour after irradiation at around a millimeter above where the sample was irradiated. This measured hydrogen concentration’s time-dependent evolution during and after a five minute irradiation was fitted to a diffusion model to determine the diffusivity of hydrogen and the radiolytic hydrogen yield in the sludge^32–37^. Essentially, the maximum concentration of dissolved hydrogen observed relates to the radiolytic yield while the time taken to reach and decay away from this maximum relates to the diffusivity. In separate experiments, the magnesium hydroxide sludge was exposed to white beam synchrotron radiation. This beam was intense enough that the rate of hydrogen generation was sufficiently large to overcome the diffusive transport away from the irradiation region, leading to a saturated solution that readily formed hydrogen bubbles. This bubble formation was imaged in these sludges with the white beam x-rays using an x-ray scintillation system^38^.

### Diffusivity

The measured effective diffusivities ($D_{ef}$) for dissolved hydrogen through the sludges, from 65 irradiation runs made with sludges of different concentrations, are plotted in Fig. 2. The diffusivity is a ratio of the measured diffusion coefficient for hydrogen and the diffusion coefficient for hydrogen in water. The comparison of these results with tortuosity models is also shown in Fig. 2. Each data point is the average value measured over an irradiation run, consisting of between 4 and 9 irradiations during which the concentration of the particulate phase was effectively constant as measured by x-ray absorption (see Experimental section for details). An example run is shown in Fig. 3 and with the fitting result tabulates in table 1. Each result in Fig. 2 is represented by an ellipse, centered on the measured effective diffusivity and particulate volume concentration with the lengths along each axis representing the associated standard error in the concentration and diffusivity, respectively. The data is presented on a log-log scale so these ellipses appear distorted.

These results, plotted in Fig. 2, were compared to the predictions from a tortuosity model developed by Beekman^39^ and a limit on tortuosity from Prager^40^. The lowest measured diffusivities lie very close to Beeckman’s model for heterogeneous catalysts^39^. The measured diffusivities are compared to this model and an upper bound on diffusivity from Prager for arbitrary shapes, endorsed by Nader and Neale in their paper on the tortuosity of spheres^40,41^. These models assume that the particles are completely impassable to hydrogen, because diffusing through a solid is much slower than diffusing through a liquid^32^. In these models, the effective diffusivity in a particular sludge is dependent only on the concentration of solid matter. Diffusion is a Brownian motion process^32,33^ and its rate is limited by the shortest available path. In heterogeneous phases, the ratio between the straight path and the average of the shortest available paths is referred to as the phase’s tortuosity^32,39–41^. The diffusivity through a tortuous phase is the diffusivity in water alone divided by the square of the phase’s tortuosity^31,40,41^. The measured diffusivities above the Prager^40^ upper bound for purely diffusive transport indicates mass transport by other mechanisms. This mass transport through water alone is affected by bulk mass motions, such as convection currents. When the particulate phase is not moving within the sludge, it dampens out these motions. Hence, in regions close to the particulate phase, diffusive transport dominates. This diffusive transport length must be at least as long as the length blocked by the particulate phase. The blocked length per unit length is equal to the volume concentration of the particulate phase ($C_{V}$). As bulk mass transport is fast, the motion will be limited by transport through the slower, diffusive, regions. Assuming infinitely fast transport through the mass transport regions (e.g. convective transport being much faster than diffusive transport^32^) and the minimum proportionality factor (i.e. 1), the upper bound on diffusion-limited motion is given by:

1 $$\begin{aligned} D_{ef} = \frac{1}{C_{V}^{2}} \end{aligned}$$

This model is also plotted on Fig. 2. All of the measured diffusivities lay below this model.

**Figure 4:**
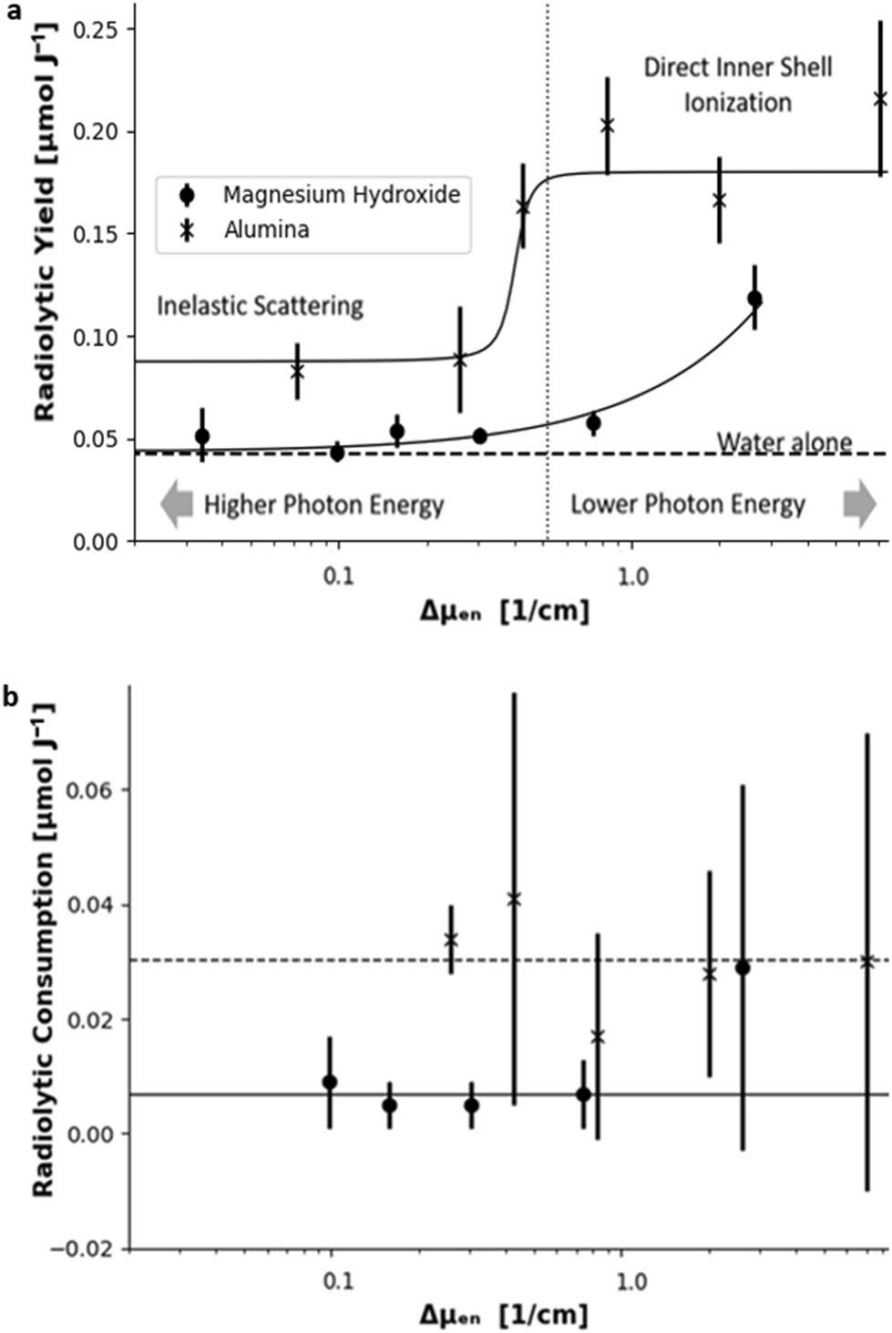
Panel (**a**) shows the measured radiolytic hydrogen yields plotted against the difference between energy absorption coefficients of the particulate phase and the aqueous phase. The highest photon energy results on this plot are measurements of radiolytic hydrogen yields from Co-60 irradiation of both magnesium hydroxide sludge and alumina sludge. The alumina measurement is taken from the literature^18^. The cross marks in the plot show the alumina sludge results and the magnesium hydroxide sludge results are plotted as solid circles. The horizontal axis is plotted on a logarithmic scale. The dashed line indicates the radiolytic yield for water alone. The vertical dotted line indicates the value at which inelastic scattering (Compton scattering) and direct ionization (photoelectric effect) cross-sections are equal in alumina; to the left of this line most ionization events are from inelastic scattering of the most loosely bound electrons; to the right of this line most ionization events are from direct photon absorption, which eject the most tightly bound electrons. The black curves indicate the trend in the data, which is an arbitrary step function for alumina and a linear fit for magnesium hydroxide. Panel (**b**) shows a plot of the measured radiolytic hydrogen consumption against the same variable but does not include the results from Co-60 irradiations. The radiolytic consumption shows no detectable trend. The dashed line plots the trend of radiolytic consumption for alumina and the solid line for trend of radiolytic consumption for magnesium hydroxide. Figure made with plots plotted with matplotlib^55^.

**Figure 5:**
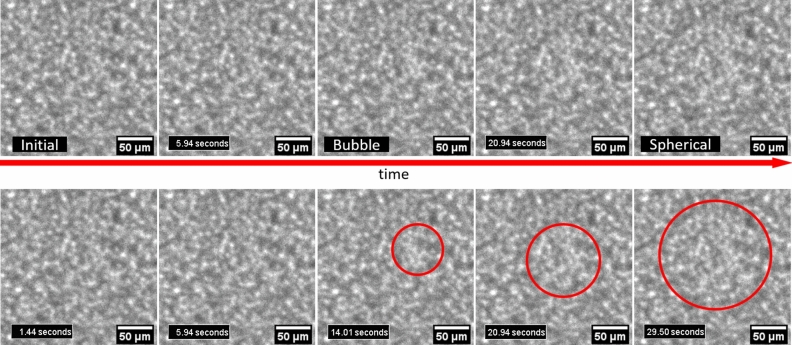
X-ray images from different times during the irradiation of a sludge-filled cuvette showing the same bubble forming. The images start from the earliest image on the left to increasingly later images on the right (1.4 s, 6 s, 14 s, 21 s, and 29.5 s with respect to the start of irradiation). The central panel shows the early signs of bubble formation around 15 s into the irradiation and the final panel shows the bubble has become spherical around 30 s into the irradiation. On the second row the images are reproduced with the approximate area considered to be occupied by the bubble marked in red to guide the eye. All images were of the same irradiation location and at the same scale. Figure made with images processed by FIJI^56^.

**Figure 6:**
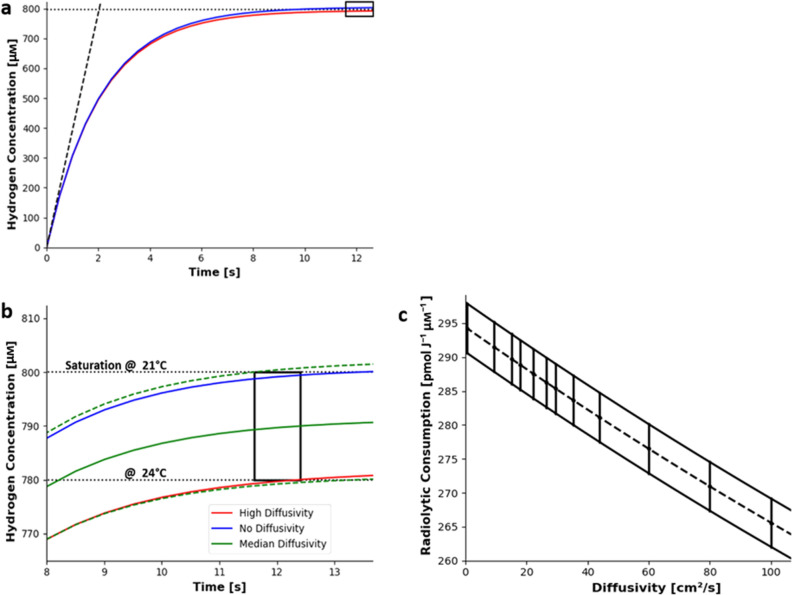
A model for hydrogen evolution in the region irradiated by white beam x-rays is plotted in panel **a**. The dotted black line indicates the hydrogen saturation concentration. The black dashed curve indicates the hydrogen formed with no transport or hydrogen consumption. The colored curves use the (288 ± 11) pmol J$^{-1}\,{\upmu }\hbox {M}^{-1}$ consumption rate. The blue curve plots the hydrogen concentration without diffusivity. The red curve plots the hydrogen concentration using the upper bound diffusivity, from equation 1. Further details are provided in the supporting material. Panel **b** (an enlargement of panel **a**) shows the same model values near the bubble formation time. The black rectangle shows the region consistent with observed bubble formation, given the possible hydrogen saturation range (from temperature uncertainty) and the observed initial bubble formation time (from shutter timing uncertainty). The green line shows the hydrogen evolution with the median diffusivity and the determined radiolytic consumption. The green dashed line indicates the hydrogen evolution with the median diffusivity, with mean consumption ± one standard error. Panel **c** shows the range of radiolytic consumption rates consistent with the observed bubble-formation time as a function of diffusivity. The range of possible radiolytic consumptions for a number of different diffusivities was calculated up to the largest diffusivity we observed in the experiments performed with monochromatic x-rays. The radiolytic consumption conforms to a linear trend at (-0.288 ± 0.004) pmolJ$^{-1}$ ${\upmu }$M$^{-1}$ cm$^{-2}$ s with intercept at (294.0 ± 0.9) pmol J$^{-1}$ ${\upmu }$M$^{-1}$ plotted with a dashed line. This defines a region consistent with observed bubble formation delimited by the black curves. Figure made with plots plotted with matplotlib^55^.

**Figure 7:**
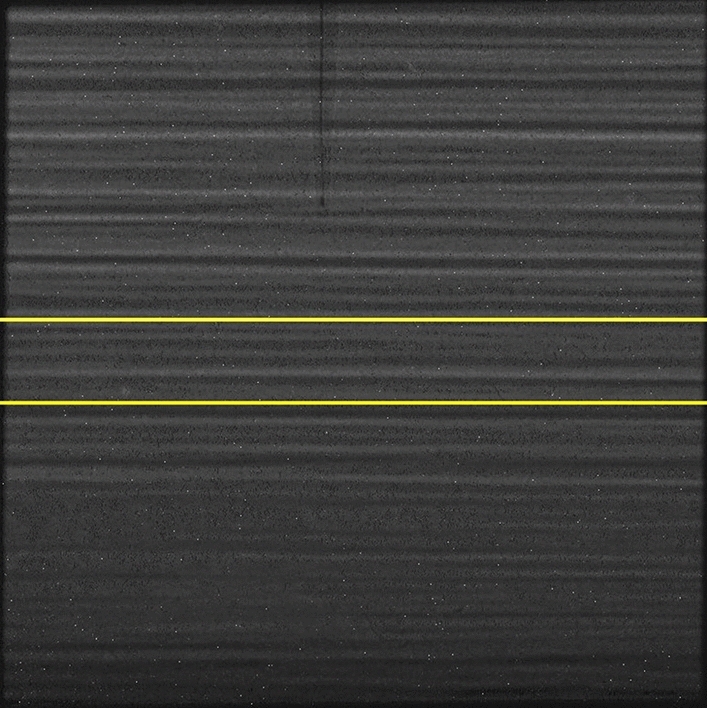
X-ray transmission image of a 5 mm $\times$ 5 mm irradiated region and the hydrogen probe tip centrally above it. Images such as this were used to determine the distance from the probe tip to the irradiated region. Yellow lines outline the region that was irradiated. Figure made with an image processed by FIJI^56^.

### Radiolytic yield

The radiolytic hydrogen yield is defined as the amount of radiolytic product produced per unit of energy deposited directly into the water present^13^. The measured radiolytic yields for hydrogen are plotted in Fig. 4. For a given sample material, each point on this plot is an amalgamation of data from every irradiation run at each particular photon energy. These measured radiolytic hydrogen yield were significantly increased for both sludge types compared to that for water alone. The results are plotted against the difference between x-ray absorption coefficients in the particulate phase and water.

This parameter was used to examine the presumption that this excess radiolytic production in the sludges when compared to radiolytic production in water alone is caused purely by energy deposition by secondary radiation. This excess radiolytic production in the sludges was assumed to be be resultant from energy being deposited into the particulate phase ($E_{solid}$) and subsequently transferring into the aqueous phase, where it drives chemical processes. These extra processes that were not present in water alone led to increased hydrogen production, measured as the increase in radiolytic yield. We assumed that the energy was transferred non-preferentially in proportion to the energy deposited into each phase, which should hold if all energy transfer occurs through secondary radiation from each phase. This assumption can be expressed as equation 2 where the increase in the radiolytic hydrogen yield ($\Delta {}G$) should be proportional to the rate of energy transfer ($\partial {}E$). If this energy transfer is performed by secondary radiation can be initiated in either phases. Hence, the net energy transfer rate is therefore proportional to the difference in energy deposited ($\Delta {}E_{solid-aq}$).

2 $$\begin{aligned} \Delta {}G \propto {} \partial {}E \propto {} \Delta {}E_{solid-aq} = E_{solid} - E_{aq} \end{aligned}$$

The energy deposited, per unit distance, in any phase is proportional to the incident x-ray radiation beam ($E_{beam}$) multiplied by the energy absorption coefficient ($\mu {}_{en}$). From this relationship it follows that the difference in energy deposited, for a unit length, can be rewritten as a function of the incident photon radiation beam energy. This difference is proportional to the difference in energy absorption coefficients ($\Delta {}\mu {}_{en} = \mu {}_{en:solid} - \mu {}_{en:aq}$) for any unit length. This factor is independent of distance so it can be applied on any length scale. Accordingly, this factor determined the increase in radiolytic hydrogen yield, provided the characteristic length scales in the sample are much smaller than the length over which the radiation is absorbed, a condition which is met in this case. Hence, the following relationships hold:

3 $$\begin{aligned} \Delta {}E_{solid-aq} = E_{beam}(x)_{en:solid} - E_{beam}(x)_{en:aq} \propto {} \Delta {}\mu {}_{en} \implies {} \Delta {}G \propto {} \Delta _{en} \end{aligned}$$

This proportionality implies that the excess radiolytic yield should be a linear function of the difference between the energy absorption coefficients for the particulate and continuous phases ($\Delta {}\mu {}_{en}$). Thus, the radiolytic yields are plotted in Fig. 4 against this parameter.

The magnesium hydroxide sludge had the expected linear relationship that appears as a smooth upward curve due to the log-linear scale used. This relationship was given by the proportionality constant (0.026 ± 0.009) ${\upmu }$mol J$^{-1}$ cm from a linear fit to the data of the form G(H$_{2}$) = (0.026 ± 0.009)$\Delta {}\mu {}_{en}$ + (0.043 ± 0.003) ${\upmu }$mol J$^{-1}$. The radiolytic yield at the intercept did not match the radiolytic yield for water alone. This residual radiolytic yield increase is likely to be a pH effect has been reported by Joseph et al^42^, because magnesium hydroxide buffers to 10.5 pH. While there was no additional scavenger of the hydroxyl radical in this system, the nanoparticle surfaces may be acting in this role. In any case, since the measurement technique concerns the earliest part of hydrogen production, its consumption by hydroxyl radicals was negligible compared to the other errors associated with the experiment.

The alumina sludge’s results show a step change in the radiolytic yield of hydrogen as a function of $\Delta {}\mu {}_{en}$. We note that this step is correlated with the switch to a dominance of Compton scattering in the direct radiation-solid interaction mechanism^13^. This is when the primary ionisation process switches between Compton scattering and the photoelectric effect. During photo-ionization a tightly-bound inner-shell electron can be released. In contrast, Compton scattering ionization generally arises from the release of a weakly-bound valence electron. After a photo-ionization, the bound electrons have to rearrange into a more energetically favorable configuration. This rearrangement produces a shower of secondary electrons called Auger electrons, all of which have a very short range. Where the step change’s inflection occurs the cross-section for Compton scattering is approximately equal to the cross-section for the photoelectric effect. Where the photoelectric effect is the dominant ionisation process, the radiolytic yield is approximately five times larger than that for water alone. Where Compton scattering dominated, the radiolytic yield is around twice that of water alone.

After repeated irradiation, the systems formed a quasi-equilibrium where the amount of hydrogen produced in the irradiated volume during irradiation is equal to the total amount diffusing away during one period (i.e., from the start of one irradiation to the start of the next one), seen as hydrogen concentration peaks converging to a near-constant value in Fig. 3. The occurrence of this quasi-equilibrium means that the net amount of hydrogen produced in an irradiation had decreased in the later irradiations. This reduction is probably because of radiolytic consumption, hydrogen present in the irradiated region, from previous irradiations, was consumed in radiation driven processes. The difference between the initial yield, in a hydrogen-free sample, and the later yield, in the irradiated samples was used to determine the radiolyic yield for hydrogen consumption. The radiolytic hydrogen consumption yield was (7 ± 4) nmol J$^{-1}$ for magnesium hydroxide and (30 ± 13) nmol J$^{-1}$ for alumina.

The radiolytic consumption of hydrogen was treated as a first-order chemical reaction in both the hydrogen present in solution and the energy deposited by irradiation. The rate constant for this reaction is estimated from the measured radiolytic consumption yields. An upper estimate of the concentration of hydrogen present during an irradiation was made. This upper estimate was divided into the consumption yield, which gave lower bound on the radiolytic consumption rate. The lower bound radiolytic consumption rate was 0.22 nmol J$^{-1}$ ${\upmu }$M$^{-1}$ for magnesium hydroxide and 0.37 nmol J$^{-1}$ ${\upmu }$M$^{-1}$ for alumina. The consumption rate was also determined from the formation of bubbles in the magnesium hydroxide sludges.

### Formation of bubbles

X-ray transmission images of the sludges irradiated with the white beam from the Diamond Light Source synchrotron captured the formation of hydrogen bubbles, as depicted in Fig. 5. This bubble formation is easier to see in a video rather than a series of still images and so an example video of bubble formation is provided in supporting information. The first hydrogen bubble formed (12.03 ± 0.13 ± 0.2 = 12.03 ± 0.4 worst case error) seconds into the irradiation. This bubble formation time was observed in an irradiation with a broad x-ray spectrum with an average 2.7 kGy s$^{-1}$ dose rate.

The error in the formation time ± 0.13 is the error in timing from irradiation start time and ± 0.2 represents error due to the shutter opening period. This irradiation start time was from when the shutter was half open. This shutter took one second to move completely up and out of the beam. In the images in the irradiation shown in Fig. 5, two fifths of the field occluded by the shutter were imaged. The first bubble formed at the top of the irradiated region so it had a shorter irradiation, due to the upward direction of shutter motion. The formation of this bubble is depicted in Fig. 5. A bubble is expected to form after the concentration of dissolved hydrogen becomes greater than the hydrogen saturation concentration. The hydrogen concentration during an irradiation was modelled including the observed hydrogen radiolytic yields (seen in Fig. 4) and diffusive transport (shown in Fig. 2), its output plotted in Fig. 6. The earliest bubble formation time is estimated to be when the hydrogen concentration reaches saturation in this model. This assumption is justified as bubbles will form at nucleation sites on nanoparticle surfaces, so it is impossible for the dissolved hydrogen in the irradiated region to stay super-saturated without bubble formation. The first bubble formation time is consistent with a radiolytic hydrogen consumption rate of (0.288 ± 0.011) nmol J$^{-1}$ ${\upmu }$M$^{-1}$. This radiolytic hydrogen consumption is an average across a broad range of photon energies.

It is noteworthy that the fractional error in this result was considerably smaller than the fractional error in the timing. This result was due to the nature of the chemical kinetics curve shown in Fig. 6. The sensitivity analysis which supports this conclusion is presented in detail in the Supplemenatry Information. Unlike the measurement of G(H$_{2}$), this measurement does concern the presence of OH and its effect on hydrogen consumption because it describes the approach of the system towards equilibrium.

The radiation induced heating was measured using a thermocouple in the same cuvette during a second irradiation, presented in Supplemenatry Information. During this irradiation a 1 K increase in temperature was observed after 100 seconds of white beam irradiation. Since the irradiation was conducted in less than this time, the effect of radiation heating the sample can be neglected.

## Discussion

These results provide insight into two different material properties. Through the diffusion results, the tortuosity of the sludges is revealed. The radiolytic yield and bubble formation results uncover the details of radiolytic production and consumption in the sludges.

The particulate phases were very tortuous, as evidenced by the data shown in Fig. 2, possibly involving nanopore networks within the particles themselves. This tortuosity is indicated by the definite tendency for the lowest diffusivities at low particulate concentrations to be observed close to Beeckman’s model^39^, since that model requires the presence of pores. The agreement between this model and the empirical data was observed, even for extremely low volume concentrations where the pore-mediated diffusion is most likely to occur on a nanoparticle-by-nanoparticle basis rather than on large interconnected pore networks.

The lowest diffusivities tend to deviate from Beeckman’s model as the particulate concentration increases. We speculate that this deviation may be due to diffusion around the particulate phase rather than diffusion through the tortuous paths within individual members of the particulate phase becoming the dominant transport route. This diffusion around the particulate phase is only possible due to the small size of the hydrogen particles.

None of the measured diffusivities above the Prager limit are above the diffusively limited transport upper bound. So, it was still meaningful to model their transport as diffusive because the flow will be limited and characterized by its slowest section, which was diffusive, validating the use of a diffusive transport model in these results.

The radiation beam was shaped into a ribbon beam (where the beam is short and wide) meaning that diffusion is effectively in only one dimension (the up-down direction). Hydrogen in this experiment is introduced by uniform irradiation. Any initial inhomogeneity due to closeness to the particulate phase surfaces was swiftly homogenised. Hence, the hydrogen is produced homogeneously in a constrained volume without stirring or other agitation. This means the method is potentially useful for future investigations into tortuosity.

The radiolytic yield results demonstrate that the mechanism for energy transfer is material dependent. Variation in the photon energy of the incident radiation for each measured yield probed the nature of that mechanism. A simple radiative transfer was observed for magnesium hydroxide since the excess yield scales linearly with $\Delta {}\mu {}_{en}$. In contrast, the results for alumina clearly demonstrate a rapid change in excess yield approximately coincident with the switch between Compton scattering dominance and photoelectric effect dominated ionization. This finding implies the involvement of Auger emission, with hydrogen either being created directly by the cascade of low-energy electrons which deposit energy near to the parent nanoparticle, or through the creation of many holes within the nanoparticle which subsequently transport into the continuous phase. Hence, the implication is that the excess hydrogen is created by a surface or near-surface effect involving Auger electrons. This implication of an Auger-electron driven process can assist in postulating a possible mechanism for the results of Reiff and LaVerne^18^. Their results show an enhanced radiolytic hydrogen production effect in the first few mono-layers of water. These results would be consistent with a short-range energy transfer mechanism, like the Auger effect seen here in alumina sludge. This effect could simply be an amplified Auger effect as irreversible electron transfer has been observed out of alumina surfaces by Chelnokov et al.^43^ Our production and consumption values for the magnesium hydroxide sludge are relevant to potential hydrogen production and bubble formation in nuclear waste sludges. The radiation in these sludges derives from the radioactive decay of radionuclides, with most activity originating from beta and gamma emitters. Thus, the effects will be similar to gamma or high-energy x-ray irradiation. The above discussion suggests that the reported intercept value (0.043 ± 0.003) ${\upmu }$mol J$^{-1}$ can be unambiguously used as the radiolytic yield for most dose rates irradiating such sludges. Using this yield and our measured consumption rate, the model predicts a steady state hydrogen concentration of (162 ± 15) ${\upmu }$M in the sludge. The radiolytic production and consumption results are not applicable to the radiation effects originating from alpha emitters. The diffusion results are independent of the chemical production process.

We have presented results that elucidate the relationship between observed macroscopic effects and the underlying radiative mechanisms in heterogeneous radiation chemical systems. These results were obtained using a novel technique for the measurement of hydrogen diffusion through a sludge. This technique allowed for the simultaneous determination of the effective diffusion coefficient and the radiolytic yield for the first time. An Auger-emission driven mechanism was inferred from the observed photon-energy dependence of the hydrogen yield in alumina sludges. This mechanism further develops previous studies of enhanced radiolytic hydrogen production on alumina surfaces^18,43^. The radiolytic hydrogen production results support the prediction of the time at which bubbles appear in a sludge.

## Methods

### Diffusion coefficient and radiolytic yield measurement technique

This experiment was typically performed with 4 to 8 cuvettes in parallel. Each cuvette was filled with a sludge. These cuvettes were placed on a linear translation stage on the synchrotron beamline, in a radiation-shielded climate-controlled room with fixed temperature (293.4 ± 0.4) K. A monochromatic x-ray beam was selected from the x-rays produced by the synchrotron with monochromator: a RuB4C double multi-layer mirror monochromator was used on B16^44–46^ and a Si(111) double crystal monochromator was used on I15^47^. The photon energies used were 20 keV, 30 keV, 40 keV, 50 keV and 60 keV. The monochromatic x-ray beam from the synchrotron was shaped into a ribbon beam (5 mm $\times$ 1 mm) using beam-defining slits which were situated upstream from the sludge-filled cuvettes. Each cuvette in turn was translated sequentially into position for irradiation and then exposed to the shaped x-ray beam for a fixed period of time. This process was repeated a fixed number of times so that each cuvette was periodically exposed to a ‘pulse’ of radiation for 5 minutes in a complete period of approximately an hour. The translations were performed slowly along a horizontal plane to minimize any disturbance to the sludges. In each cuvette, an electrochemical hydrogen probe (Unisense A/S, Aarhus, Netherlands)^30^ was held approximately 1 mm above the irradiated region of the sludge and measured the hydrogen concentration over time at that position. A Canberra PD300-500CB photo-diode (Canberra, United States), calibrated by the Physikalisch-Technische Bundesanstalt, was used to determine the power deposited into the sample during irradiation^48,49^. After irradiation, x-ray images were taken of the probes inside the filled cuvettes in-situ from two orthogonal directions. These images were used to determine the exact height of the probe tip relative to the centre of the irradiated region and the depth of the probe into the cuvette, as shown in the x-ray image in Fig. 7. The height of the probe was a key parameter in the hydrogen diffusion model. The depth of the probe along the beam axis during the ribbon beam irradiation was used to calculate a correction factor from the global energy deposition to the local deposition under the probe. The hydrogen radiolytic yield and diffusivity were determined from fitting, with a Levenberg-Marquardt algorithm, the measured hydrogen concentration to a one-dimensional diffusion model^33–37,49^, details of which are given in the supporting information. The best fit parameters of this model gave the diffusion coefficient (cm$^{2}$ s$^{-1}$) and hydrogen concentration increase rate (${\upmu }$M s$^{-1}$). The hydrogen concentration increase rate was divided by the power absorbed by the water fraction of the sludge (${\upmu }$W), and multiplied by the volume of sludge’s water fraction (${\upmu }$L). This calculation produces the radiolytic hydrogen yield in the sludge (${\upmu }$mol J$^{-1}$). The rate of hydrogen consumption can be estimated as the ratio of these two quantities, i.e., consumption is equal to the radiolytic consumption yield divided by the amount produced. The radiolytic yield for consumption was calculated from the difference in the radiolytic yields between the first and last irradiations, in nmol J$^{-1}$. The amount of hydrogen produced by an irradiation was estimated directly from the first irradiation step because the sample is initially hydrogen-free. This method allows us to compare the system in a quasi-steady state with the initial (hydrogen-free) system. This method was not used to produce the results from Co-60 irradiation, which were extracted from available literature^18,50^ where gas chromatography was employed to measure the hydrogen concentration after irradiation.

### Bubble formation imaging

Bubble formation in magnesium hydroxide sludges was imaged during a white beam irradiation. A 10 mm-wide sludge-filled cuvette was exposed to the full output from the B16 bending magnet source^46^. Hence this type of irradiation is called white beam irradiation because it comprises of a broad range of x-ray wavelengths. This white beam was shaped into a 2 mm $\times$ 2 mm beam with in-vacuum slits. The x-rays transmitted through the cuvette were imaged with a scintillator-based detector designed for use with white beam^38^, placed directly behind the cuvette. The white beam spectrum from the bending magnet source was simulated with X-ray Oriented Programs 2.44^51^. The simulated spectrum allowed the determination of the power deposition into the water fraction of the sludge as function of photon energy. From this power deposition, the hydrogen production rate, in mol s$^{-1}$, was calculated using the observed linear relationship for radiolytic yields in magnesium hydroxide sludges to calculate a hydrogen yield per unit volume. From this yield, the hydrogen consumption (i.e. the hydrogen concentration and deposited power multiplied by the radiolytic consumption rate) was subtracted to produce an effective yield. This effective yield was multiplied by the known irradiated water volume (36 ${\upmu }$L) to produce the hydrogen production rate. These rates were inserted into radiation-driven hydrogen concentration evolution model. This model was an ordinary differential equation representing the kinetics of radiolytic hydrogen production and consumption^13^. An explicit finite difference 2D diffusion model^33^ was used to determine the influence of diffusion, which had a substantially weaker effect than radiolytic consumption. The model’s details are given in the Supplemenatry Information. This model determined an expected time when the solution was saturated with dissolved hydrogen. This time was considered to be the earliest time for hydrogen bubble formation. This expected bubble formation time was compared to the observed earliest bubble formation time. The consumption rate that gave a hydrogen saturation time equal to the observed bubble formation time was reported above. Mass transport was considered unimportant for these processes because the characteristic time to diffuse significantly outside (1 mm) of the irradiated region was greater than 100 seconds. Bubble formation was observed much earlier than this characteristic time.

### Sludge preparation

Most samples used in this work were simple mixtures of water and solid nanoparticles. The nanoparticles used to prepare these mixtures were: magnesium hydroxide nanoparticles less than 100 nm diameter (Sigma Aldrich, UK)^52^; magnesium hydroxide nanoparticles 10 nm diameter (US Research Nanomaterials, USA)^53^; and aluminium oxide nanoparticles less than 50 nm diameter (Sigma Aldrich, UK)^54^. Each mixture was prepared in a beaker and then transferred into the cuvettes. A few grams of nanoparticles were weighed out and ultra-pure water was then added to the beaker. The mixture was stirred until a uniform consistency was achieved and then transferred into cuvettes, which had a small volume of water added prior to this transfer, to avoid trapping air bubbles in the sludge (x-ray imaging was used to confirm that no air bubbles were trapped before the experiments proceeded). In the cuvettes, the mixture was allowed to settle and the solids concentrated in the lower half of the cuvette. The mixture in this lower half of the cuvette formed the sludge used in our experiments. This work also used corroded magnesium sludge, i.e. the material obtained from the corrosion of metallic magnesium in a heated tank over a number of years to simulate corroded cladding sludges found in spent nuclear fuel legacy ponds^26,27^. The corrosion processing was performed by the National Nuclear Laboratory, UK, and was kindly donated for this work by Sellafield Ltd. The type of nanoparticle used in each experimental run is tabled in supporting information. The type of nanoparticle used in each experimental run is tabled in supporting information.

## Supplementary information

Supplementary Video.

Supplementary Information.
