# Supplementary material for: First observation of radiolytic bubble formation in unstirred nano-powder sludges and a consistent model thereof
Source: Sci Rep. 2021 Nov 24;11:22882. doi: 10.1038/s41598-021-01868-1 (PMC8613212; doi:10.1038/s41598-021-01868-1)
Supplement: Supplementary file 2 — Supplementary Information. [file 41598_2021_1868_MOESM2_ESM.docx]

First Observation of Radiolytic Bubble Formation in Unstirred Nano-powder Sludges and a Consistent Model Thereof (Supplementary Information)

Mel O’Leary*, Aliaksandr Baidak, Martyn Barnes, Thomas Donoclift, Christopher Emerson, Catarina Figueira, Oliver Fox, Annette Kleppe, Aaron McCulloch, Darryl Messer, Robin Orr, and Fred Currell

Included here are the supplementary experimental details and a table of all the results used in the paper. Material presented here includes excerpts from Mel O’Leary’s PhD thesis^1^ for completeness.

S1 General Experimental Setup

X-ray radiation produced, as described below in section S1.1, at the Diamond Light Source (a synchrotron x-ray source)^2–4^ was used to irradiate various samples. Irradiations were mostly carried out with monochromatic x-rays. The photon energy was selected using a monochromator.^5–8^ After the monochromator, the x-ray beam was shaped into the required dimensions using slits. The sample and a photo-diode were placed sequentially in the beam path as illustrated schematically in figure S1. The timing of the x-ray beam irradiation was controlled by opening and closing a shutter. The beam intensity was varied with aluminum attenuators.

The dose rate was set through selection of an aluminum filter, thicker filters leading to greater attenuation and a lower dose rate. The length of exposure to radiation, and hence the total dose deposited was determined by opening the shutter for a specific time under computer control. The x-ray beam power was determined using a diode calibrated by the Physikalisch-Technische Bundesanstalt, which was a Canberra PD300-500CB photo-diode (Canberra, United States).^9^

S1.1 *Synchrotron Radiation Production*

Monochromatic x-rays were generated at the Diamond Light Source synchrotron, on beamlines I15 and B16.^5–8^ In this work a wiggler insertion device, on I15,^8^ and a bending magnet insertion device, on B16,^5–7^ produced the x-rays used for irradiation of the samples. The x-rays were generated with a broad spectrum by electrons being accelerated by a magnetic field (relativistic magneto-bremsstrahlung).^2–4^ Electrons in the Diamond Light Source storage ring are accelerated up to 3 GeV kinetic energy (γ ≈ 6000).^3^ In ordinary operation mode, the electrons are grouped into 900 bunches of 0.6 nC (3.75 × 10^6^ electrons), each bunch ≈ 13 mm long at full width half maximum (FWHM) intensity in their direction of travel. The radiation emitted by these bending magnets has a critical energy of 8.4 keV.^6^ The I15 wiggler critical energy is 20.9 keV.^8,10,11^ The brilliance of the Diamond Light Source beamlines is 10^16^ photons (s mm^2^ mr^2^ 0.1% BW)^-1^ for a bending magnet source and 10^18^ photons (s mm^2^ mr^2^ 0.1% BW)^-1^ on a wiggler source.^4^

X-rays of a specific wavelength were selected with a monochromator.^5–8^ A RuB4C double multilayer mirror monochromator was used on B16.^5–8^ A Si(111) double crystal monochromator (DCM) was used on I15.^8^ The samples were positioned upstream of a calibrated Canberra PD300-500CB photo-diode.^9^ This photo-diode measured the instantaneous power in the x-ray beam, after traveling through the sample, during irradiations. These measurements were used for dosimetry as described below. The photo-diode measurements gave the power in the x-ray beam attenuated by the sample, during each irradiation. Photo-diode measurements were taken from irradiation of the cuvette container without the sample. Together these measurements were used to calculate the rate of energy deposition during each irradiation.

S2 X-ray Dosimetry and Metrology

The dosimetric methods used in this project are described below. These methods were used to calculate, from the photo-diode measurements taken during irradiations, the amount of energy deposited in a sample. The photo-diode measured the current produced from ionizations by the x-ray beam in a 300 mm^2^ circular area of implanted partially depleted planar silicon, which was 500 µm-thick. The diode calibration correlates the power of the x-ray beam to the current produced. This correlation was called the sensitivity of the diode. The photo-diode was connected to an amplifier which converted the current into a voltage at a given gain. This voltage was used to determine the rate of energy deposition by the x-rays in the samples.

The rate of energy deposition in the sample by the x-ray beam was dependent on the power of the incident x-ray beam. Photo-diode measurements were used to determine this incident power. The sensitivity of the calibrated photo-diode (i.e., the calibrated ratio of current through the diode to the power in the x-ray beam traveling through the photo-diode) was divided into the photo-diode current measurements to produce the x-ray beam power at time *t*. This x-ray beam power was attenuated by the sample (with attenuation coefficient µ and thickness *x*), the sample container back-window (with attenuation coefficient and thickness *z*), and the air (with attenuation coefficient µ_air_) and a thickness *d* (i.e. the distance between the diode and the sample container). The incident x-ray beam power was given by the attenuated x-ray beam power measured divided by the amount of attenuation. The rate of energy deposition in the sample (with energy absorption coefficient µ*_en_*) was calculated from this incident power by multiplying by the energy absorption of the sample. Bringing these factors altogether gives equation S2.1, allowing the calculation of the rate of energy deposition *P_dep_*(*t*).

$P_{dep}\left( t \right)= \frac{1-e^{-\mu_{en}x}}{e^{-\mu x- \mu_{window}z- \mu_{air}d}}\frac{M_{Sample}(t)}{S}$, equation S2.1

Where *M_Sample_(t)* is the photo-diode measurement taken during irradiation while the sample was in the cuvette, *S* was the product of the photo-diode sensitivity and the gain of the amplifier used in photo-diode current digitization. The fraction of these two was the attenuated x-ray beam power as discussed above.

S2.1 *Determining Sample Thickness from X-ray Attenuation*

The thickness of each sample was determined from the ratio of photo-diode measurements with (*M_with_*) and without that sample, as described in equation S2.2. These measurements were divided by the equivalent incident x-ray beam power measurement (*M_equiv_*) if the sample were not present, calculated from equation S2.3. This equivalent measurement was calculated from measurements when the sample was not present and the beam was attenuated by air instead. As the incident x-ray beam power was directly proportional to the storage ring current (*I_ring_ (t)*). This relationship is shown in figure S2 which plots attenuated x-ray beam power against coincident storage ring current.

The photo-diode measurements without this sample instead of the sample and do not necessarily have equivalent incident x-ray beam power. These measurements without the sample were used with the simultaneous current in the synchrotron ring (*I*_ring_) to calculate the average ratio from.

$x= \frac{\ln\left( \left\langle\frac{M_{with}}{M_{equiv}} \right\rangle\right)}{\mu- \mu_{air}}$, equation S2.2

$M_{equiv}\left( t \right)=\left\langle\frac{M_{air}}{I_{ring}} \right\rangle I_{ring}(t)$, equation S2.3

S2.2 *Rate of Energy Deposition During Pulses*

When considering the effects of dose rate upon samples it is important to differentiate between the instantaneous dose rate and the average dose rate since the synchrotron is a pulsed source.^3,4^ The pulsed nature of the source means that for most of the irradiation time the sample was not being irradiated. This section describes how the average rate of energy deposition during these pulses was calculated. This average was derived from the effective irradiation time during the pulses. The ratio of the total irradiation time to the effective irradiation time was multiplied with the measured rate of energy deposition to give the deposition rate during pulses.

The synchrotron ring current is stored in 900 bunches which complete a cycle every 1.872 µs.^3^ Each bunch produces an x-ray pulse when the electrons pass through the bending magnet, wiggler or undulator source.^2–4,10,11^ The rate of energy deposition measured with the photo-diode was averaged over the whole cycle. The whole cycle was equivalent to the total irradiation time discussed above. The time structure of the radiation pulses measured with a streak camera is shown in figure S3. The pulse shape was approximately Gaussian with a 43.4 ps FWHM period. This FWHM time was equivalent to the effective irradiation time discussed above. This equivalence holds, although the rate of energy deposition during each pulse varies over time, and is directly proportional to the instantaneous beam power. The average power can be considered to be the power during a top-hat pulse. The duration of these FWHM periods over the whole cycle (39.1 ns) gave the time for energy deposited over the whole cycle at this average energy deposition rate during a pulse. This time was used to calculate the ratio of the irradiation time to pulse time (47.9 (s/s)), which was multiplied by the photo-diode measured rate of energy deposition to get the average energy deposition rate during each pulse.

S2.4 *Determining Concentrations from X-ray Attenuation*

Presuming the sample was purely a mixture of two chemicals with x-ray attenuation coefficients µ*_Substance_*_1_ and µ*_Substance_*_2_, then the (w/w) concentrations of these substances (*C*_Substance1_, *C*_Substance2_) were calculated from the sample’s x-ray attenuation coefficient (µ) using equation S2.4. This attenuation coefficient was determined, using equation S2.5, from the photo-diode measurements from x-ray beam attenuated by a sample-filled container (*M_Sample_*) and thier equivalent measurement (*M_equiv_*), as defined in equation S2.3. Equation S2.5 effectively reverses equation S2.2 to get an unknown x-ray attenuation coefficient from a known thickness. The photo-diode measurements of the x-ray beam attenuated by a water filled sample container, gave the sample container thickness (*x_samplecontainer_*) using equation S2.2. In the cuvettes, this distance was the same when it was filled with any sample given its rigid nature.

$C_{Substance2}=1-C_{Substance1}= \frac{\mu-\mu_{Substance1}}{\mu_{Substance2}-\mu_{Substance1}}$, equation S2.4

$\mu= \frac{\ln\left( \frac{M_{Sample}}{M_{equiv}} \right)}{x_{samplecontainer}}+ \mu_{air}$, equation S2.5

S3 Hydrogen Diffusion Model for Fitting to Measured Hydrogen Concentration

The irradiation produced radiolytically-generated molecular hydrogen from water, which diffused away from the irradiated region. Hydrogen mass transfer in water usually happens by bulk motion, e.g., from convection.^12^ Hydrogen mass transfer was primarily due to diffusion, but only when these bulk water motions are dampened.^12,13^ Under these circumstances, the hydrogen concentration ([*H*_2_]) is governed by the diffusion equation, equation S3.1 in which *D* is the diffusion coefficient for the diffusate (hydrogen) in the diffusive medium (in this case the sludge mimic).^12,13^

$\frac{d\left[ H_{2} \right]}{dt}= \nabla\cdot(D\nabla\left[ H_{2} \right])$ , equation S3.1

The experimental geometry was deliberately chosen to give a solution which was well approximated by an analytical form. Given that the irradiated region was long in two orthogonal horizontal directions (i.e., along the beam axis and perpendicular to the beam axis) diffusion in this geometry can be approximated as diffusing planes that travel vertically. Hydrogen diffused from an instantaneous production event and was described by the sum of two error functions separated by half the height of the irradiated region,^13^ denoted *h*. Irradiation was not instantaneous so this solution must be integrated over the duration of the irradiation (*T_irrad_*) as shown in equation S3.2. The hydrogen concentration from an irradiation event was fitted to this function. There was a series of irradiations into each cuvette. The hydrogen concentration after an irradiation event was a combination of hydrogen produced in that irradiation and from previous irradiations. This background hydrogen concentration was extrapolated from the concentration variation just before an irradiation event.

$\left[ H_{2} \right]\left( x, t \right)=\left[ H_{2} \right]_{inSecond}\int_{0}^{T_{irrad}} \left( \mathrm{erf} \left( \frac{x+h}{\sqrt{4D\left( t-\tau\right)}} \right)+\mathrm{erf} \left( \frac{h-x}{\sqrt{4D\left( t-\tau\right)}} \right) \right)d\tau$, equation S3.2

In equation S3.2 [*H*_2_]*_inSecond_* was the dissolved hydrogen concentration increase rate during one second of irradiation. The dissolved hydrogen concentration was multiplied by the water volume in the irradiated region to get a hydrogen production rate. This hydrogen production rate ([*H_2_*]*_inSecond_*) and the diffusion coefficient (*D*) were adjustable parameters used in equation S3.2 to correlate with the measured concentration trend.^14–17^ The hydrogen production rate was divided by the rate of energy deposition into the fraction water of the sludge mimic to give the radiolytic hydrogen yield. The average rate of energy deposition, measured with the photo-diode, was multiplied by a factor accounting for power deposition variation through the cuvette, see equation S3.3. In this equation, *x_probe_* was the distance from the cuvette edge to the tip of the probe. The x-ray absorption coefficient of the sludge mimic was denoted by µ*_sludge_*.

$\frac{e^{-\mu_{sludge}x_{probe}}}{\left\langle e^{-\mu_{sludge}x} \right\rangle}$, equation S3.3

S4 Bubble Formation Model

The analysis presented above presumes a monochromatic x-ray source. The Diamond Light Source synchrotron operates with 300 mA, 3 GeV electrons in 900 separate bunches.^3^ At the B16 beamline B16 a broad spectrum of x-rays are produced by the bending magnet source. ^5,8^ This broad spectrum was simulated, between 100 eV and 100,000 eV, with the XOP v2.4 (X-ray Oriented Programs) software package,^18^ for a bending magnet source tuned to 1.35 T. This simulated spectrum (*S*(*E*)), in W keV^-1^ as a function of photon energy (*E*), was then used as a source term for modelling the energy deposition into the sludge mimics. This energy was deposited by a beam shaped into a 2 × 2 mm square. This beam shape was equivalent to a 50 × 50 µradians square divergence of the synchrotron x-ray beam. This beam was attenuated by the side of the cuvette, which has a x-ray attenuation coefficient (µ*_glass_(E)*) and about 1 mm thickness (*x_edge_*). This energy deposition, as a function of photon energy (*D*(*E*)), was calculated with the NIST x-ray attenuation and energy absorption coefficients (µ*_en_(E)*),^19^ see equation S4.1. The radiolytic yield as a function of photon energy (*G*(*E*)) was extrapolated from the measured relationship, see section 2.2 in the paper. The energy deposition was multiplied with the radiolytic yield, producing the amount of hydrogen produced per second as a function of photon energy (µmol keV^-1^). This hydrogen production rate density spectrum was divided by the volume of the irradiated region to calculate the hydrogen concentration increase rate, in the irradiated region, as a function of photon energy, as plotted in figure S4. This hydrogen concentration increase rate density was integrated across all photon energies, to produce a total hydrogen concentration increase rate (µm s^-1^). This total, together with the extrapolated radiolytic yield function, was calculated to be 390 ± 90 µm s^-1^, which gave a minimum time to saturation of 2.1 ± 0.5 s, for a 13 ± 10% (w/w) magnesium hydroxide sludge mimic. Also calculated was the value for a constant radiolytic yield function equal to the intercept value. This function yields a total production rate of 77 ± 8 µm s^-1^, which gives a minimum time to saturation of 10.2 ± 0.9 s, for a 13 ± 10% (w/w) magnesium hydroxide sludge mimic.

$D\left( E \right)= e^{-\mu_{glass}(E)x_{edge}}\left( 1-e^{-\mu_{en}(E)x} \right)S(E)$, equation S4.1

$\frac{d\left[ H_{2} \right]}{dt}=G_{H}- \left[ H_{2} \right]C_{H}$, equation S4.2

This production rate (*G_H_*) was used as a source term for a numerical simulation of hydrogen production and consumption. The radiolytic consumption rate (*C_H_*) was determined with this model. These processes were represented by the differential equations in equation S4.2. The differential equations where solved by fourth order Runge-Kutta, with a 10 µs time step.

The radiolytic consumption rate was dependent on the hydrogen concentration present during irradiation. The average radiolytic consumption rate for each measured radiolytic consumption is plotted in figure S5. These plotted rates were determined with average hydrogen concentrations during irradiation. The average concentrations were the determined radiolytic yields multiplied by the average dose rates in water. This average increase in hydrogen concentration was divided into the radiolytic consumptions, plotted in figure S4, to produce the rates, plotted in figure S5. These consumptions give an average range from 0.22 to 0.7 nmol J^-1^ µm^-1^.

The simulation was extended to investigate the effect of diffusion production rate. For this, the simulation was transformed into a coarse 2-dimensional explicit finite difference model of hydrogen production and diffusion,^13^ for the sludge mimic in the cuvette. The elements in the model were 2 × 2 mm even blocks in the cuvette, which was treated as a 5 × 5 element box. The top edge of the simulated cuvette was treated as absorbing and the other cuvette edges were treated as reflecting. The top boundary edge represented the sludge-atmosphere interface where hydrogen can escape to atmosphere, and the other edges were sludge-glass interfaces. This simulation was performed using an upper bound coefficient which was higher than the highest observed diffusion coefficient for the magnesium hydroxide sludge, given the results presented in figure 2. The model concentration of the central element of these models is plotted in figure 6 in the paper. The curve where the diffusion coefficient was zero in the model is equivalent to the original numerical solution to the differential equations.

These consumption rates were included in the model as independent of photon energy, as the average consumption rates appear to be in figure S5. The model preformed with the upper bound consumption rate appears to come to a steady state before saturation and with the lower bound consumption rate the solution saturates in the model after 4.21 ± 0.08 seconds. The radiolytic consumption rate which produced saturation in the model values consistent with the observed initial bubble formation time was 0.288 ± 0.011 nmol J^-1^ µm^-1^. This consumption rate was the average rate that produced saturation immediately before the appearance of the first observed bubble. This average value was between the rates given bubble appearance time minus errors 11.6 seconds and plus errors 12.4 seconds, where the hydrogen saturation upper bound was 800 µm and the lower bound was 780 µm,^20^ which overestimates the range of possible saturation concentrations, with and without diffusion, eight distinct values in total. The error was determined from a statistical spread of these values.

These considerations require there to be negligible radiative heating. This was the case because it took over 50 seconds to induce at least a 0.2 K temperature increase. The temperature during an irradiation event was measured in the cuvette by inserting a thermocouple into the region of the sample irradiated. The results from this measurement are reported in table S2. This irradiation was performed after the bubble formation experiment on a region 2.4 cm above where the first irradiation was incident.

FIGURES


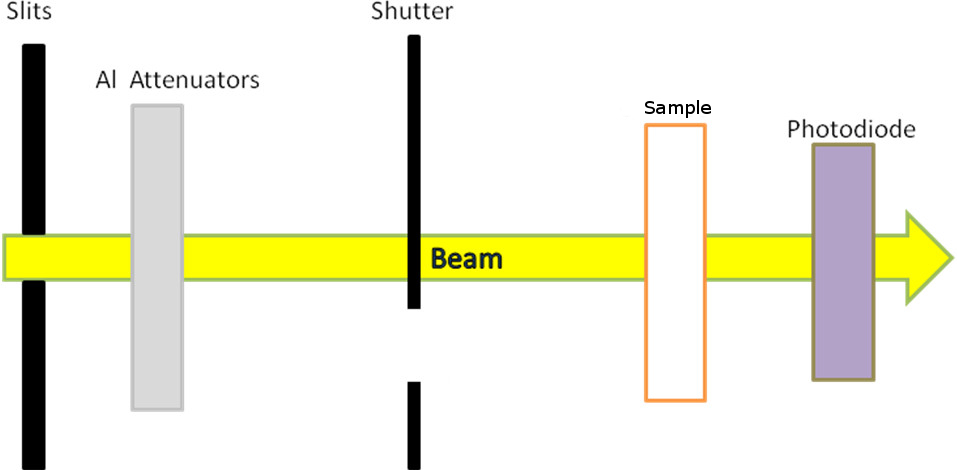


**Figure S1**. Schematic representation of the general setup for irradiation experiments. The yellow arrow represents the path of the x-ray beam. First, the beam was shaped by beam-defining slits and attenuated by aluminum foils. The beam was allowed along the beamline by a shutter controlled by the automated experimental system. The sample to be irradiated and the photo-diode were placed further downstream.


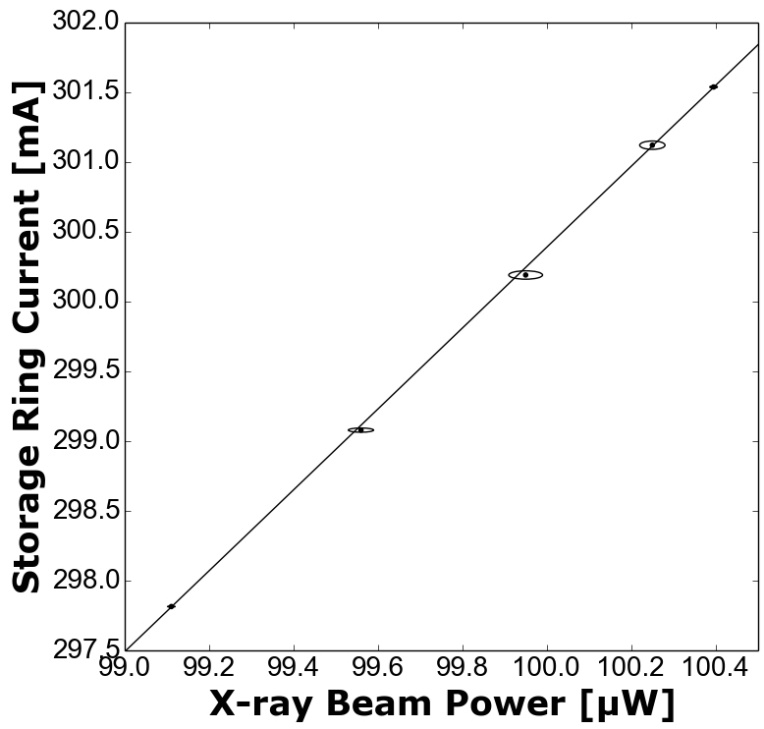


**Figure S2** Plot of concurrent storage ring current and the x-ray beam power determined using a photodiode. The linear proportionality between the two measurements, justifies the methods used in equation S2.3.


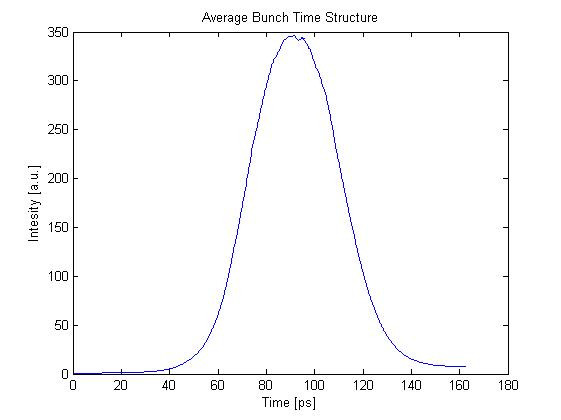


**Figure S3.** Electron bunch pulse shape measured by streak camera. Gaussian pulse profile with centre at 98 ps and a 43.4 ps FWHM bunch period.


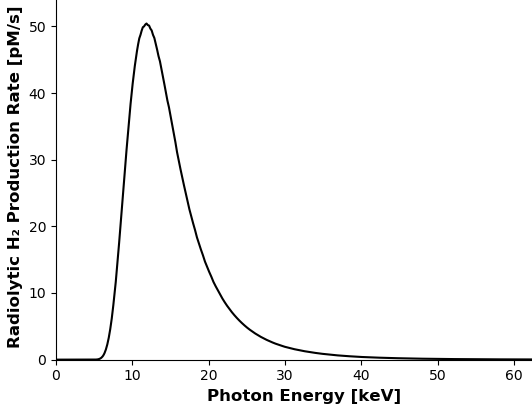


**Figure S4.** Simulated hydrogen concentration increase rate, in the irradiated region, as a function of photon energy.


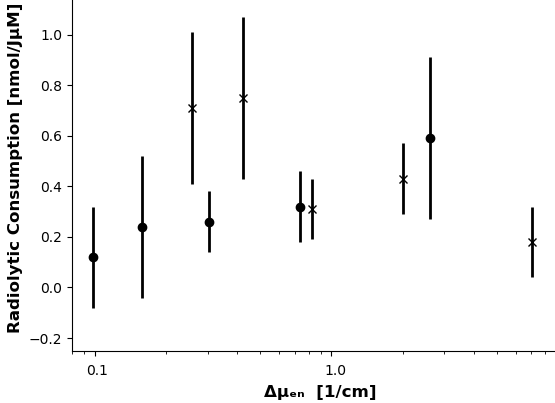


**Figure S5.** The measured radiolytic hydrogen consumption rates as a function of Δµ_en_, from figure 4. The radiolytic consumption rates in this figure were the radiolytic consumptions shown in figure 4 divided by the average amount of hydrogen produced in an irradiation.

TABLES.

**Table S1.** Table of all the results used in paper. The nanoparticles used in this paper were US Research Nanomaterials Inc. magnesium hydroxide 10 nm, Sigma Aldrich >100 nm magnesium hydroxide, and Sigma Aldrich >50 nm aluminum oxide. Corroded magnesium sludge (CMgS) was also used.

| Nanoparticle Type | Photon Energy (keV) | | Probe Height [mm] | Concentration  (w/w) | Concentration  (v/v) | Dose [Gy] | Dose Rate [Gy s^-1^] | Radiolytic Yield [µmol J^-1^] | Effective Diffusivity  (D/D) | Estimated  Covariance |
| --- | --- | --- | --- | --- | --- | --- | --- | --- | --- | --- |
| US Research Nanomaterials Inc. Magnesium Hydroxide 10 nm | | 20 | 0.89 ± 0.03 | 0.172 ± 0.018 | 0.09 ± 0.009 | 660 ± 60 | 105 ± 9 | 0.102 ± 0.010 | 0.684 ± 0.003 | 0.006 ± 0.003 |
| US Research Nanomaterials Inc. Magnesium Hydroxide 10 nm | | 20 | 1.05 ± 0.03 | 0.205 ± 0.003 | 0.1121 ± 0.0016 | 380 ± 40 | 60 ± 6 | 0.146 ± 0.015 | 0.6719 ± 0.00515 | 0.0044 ± 0.0011 |
| US Research Nanomaterials Inc. Magnesium Hydroxide 10 nm | | 20 | 1.05 ± 0.03 | 0.205 ± 0.003 | 0.112 ± 0.0017 | 380 ± 40 | 60 ± 6 | 0.17 ± 0.02 | 1.243 ± 0.0495 | 0.005 ± 0.003 |
| US Research Nanomaterials Inc. Magnesium Hydroxide 10 nm | | 20 | 0.85 ± 0.03 | 0.187 ± 0.003 | 0.0997 ± 0.0014 | 480 ± 50 | 77 ± 8 | 0.096 ± 0.0096 | 0.522 ± 0.013 | 0.0020 ± 0.0014 |
| US Research Nanomaterials Inc. Magnesium Hydroxide 10 nm | | 20 | 0.76 ± 0.03 | 0.1923 ± 0.0018 | 0.1032 ± 0.0009 | 630 ± 60 | 101 ± 9 | 0.086 ± 0.009 | 0.606 ± 0.008 | 0.0050 ± 0.0009 |
| US Research Nanomaterials Inc. Magnesium Hydroxide 10 nm | | 20 | 1.05 ± 0.03 | 0.214 ± 0.003 | 0.1175 ± 0.0016 | 370 ± 40 | 59 ± 6 | 0.071 ± 0.007 | 0.7498 ± 0.0055 | 0.0019 ± 0.0008 |
| US Research Nanomaterials Inc. Magnesium Hydroxide 10 nm | | 20 | 1.23 ± 0.03 | 0.216 ± 0.002 | 0.1191 ± 0.0011 | 380 ± 40 | 61 ± 6 | 0.11 ± 0.0112 | 0.509 ± 0.009 | 0.0021 ± 0.0003 |
| US Research Nanomaterials Inc. Magnesium Hydroxide 10 nm | | 20 | 0.85 ± 0.03 | 0.195 ± 0.003 | 0.1053 ± 0.0015 | 480 ± 50 | 76 ± 8 | 0.11 ± 0.0111 | 0.723 ± 0.007 | 0.0038 ± 0.0008 |
| CMgS | | 20 | 0.74 ± 0.03 | 0.1019 ± 0.0015 | 0.0494 ± 0.0007 | 670 ± 60 | 105 ± 9 | 0.14 ± 0.03 | 2.8 ± 0.55 | 2 ± 3 |
| CMgS | | 20 | 1.05 ± 0.03 | 0.118 ± 0.002 | 0.0586 ± 0.0007 | 390 ± 40 | 63 ± 6 | 0.22 ± 0.03 | 2.2 ± 0.3 | 0.15 ± 0.10 |
| CMgS | | 20 | 1.05 ± 0.03 | 0.1186 ± 0.0016 | 0.0587 ± 0.0008 | 410 ± 40 | 66 ± 7 | 0.13 ± 0.02 | 1.6 ± 0.3 | 0.017 ± 0.011 |
| CMgS | | 20 | 0.75 ± 0.03 | 0.114 ± 0.002 | 0.05599 ± 0.0011 | 480 ± 50 | 79 ± 8 | 0.169 ± 0.017 | 1.5 ± 0.2 | 0.016 ± 0.007 |
| CMgS | | 20 | 0.74 ± 0.03 | 0.1045 ± 0.0015 | 0.0508 ± 0.0007 | 670 ± 60 | 105 ± 9 | 0.112 ± 0.011 | 0.9314 ± 0.0098 | 0.49 ± 0.14 |
| CMgS | | 20 | 1.05 ± 0.03 | 0.128 ± 0.002 | 0.064 ± 0.0012 | 390 ± 40 | 63 ± 6 | 0.182 ± 0.018 | 1.034 ± 0.006 | 0.09 ± 0.03 |
| CMgS | | 20 | 1.05 ± 0.03 | 0.1318 ± 0.0017 | 0.0662 ± 0.0008 | 410 ± 40 | 65 ± 7 | 0.111 ± 0.011 | 0.75 ± 0.04 | 0.0045 ± 0.0008 |
| CMgS | | 20 | 0.75 ± 0.03 | 0.116 ± 0.002 | 0.0575 ± 0.0011 | 480 ± 50 | 79 ± 8 | 0.121 ± 0.012 | 0.92 ± 0.025 | 0.028 ± 0.010 |
| Sigma Aldrich Magnesium Hydroxide | | 20 | 0.94 ± 0.03 | 0.631 ± 0.003 | 0.497 ± 0.003 | 480 ± 50 | 76 ± 8 | 0.109 ± 0.011 | 0.48 ± 0.045 | 0.015 ± 0.003 |
| Sigma Aldrich Magnesium Hydroxide | | 20 | 1.25 ± 0.03 | 0.652 ± 0.006 | 0.522 ± 0.005 | 270 ± 30 | 44 ± 4 | 0.24 ± 0.06 | 0.398 ± 0.01 | 0.04 ± 0.03 |
| Sigma Aldrich Magnesium Hydroxide | | 20 | 1.23 ± 0.03 | 0.599 ± 0.004 | 0.461 ± 0.003 | 300 ± 30 | 47 ± 4 | 0.069 ± 0.007 | 0.395 ± 0.0095 | 0.003 ± 0.002 |
| Sigma Aldrich Magnesium Hydroxide | | 20 | 0.85 ± 0.03 | 0.692 ± 0.006 | 0.57 ± 0.005 | 340 ± 30 | 55 ± 5 | 0.153 ± 0.015 | 0.387 ± 0.0055 | 0.008 ± 0.003 |
| Sigma Aldrich Magnesium Hydroxide | | 20 | 0.94 ± 0.03 | 0.655 ± 0.004 | 0.525 ± 0.003 | 470 ± 50 | 75 ± 8 | 0.122 ± 0.012513 | 0.39 ± 0.035 | 0.006 ± 0.003 |
| Sigma Aldrich Magnesium Hydroxide | | 20 | 1.25 ± 0.03 | 0.665 ± 0.005 | 0.536 ± 0.005 | 260 ± 30 | 42 ± 4 | 0.146 ± 0.015 | 0.386 ± 0.016 | 0.0011 ± 0.0002 |
| Sigma Aldrich Magnesium Hydroxide | | 20 | 1.23 ± 0.03 | 0.630 ± 0.004 | 0.497 ± 0.003 | 290 ± 30 | 46 ± 4 | 0.097 ± 0.009 | 0.38 ± 0.01 | 0.0018 ± 0.0008 |
| Sigma Aldrich Magnesium Hydroxide | | 20 | 0.85 ± 0.03 | 0.721 ± 0.006 | 0.606 ± 0.005 | 330 ± 30 | 52 ± 5 | 0.104 ± 0.0105 | 0.384 ± 0.01 | 0.047 ± 0.015 |
| Sigma Aldrich Magnesium Hydroxide | | 20 | 1.14 ± 0.03 | 0.3677 ± 0.0004 | 0.2343 ± 0.0003 | 345 ± 4 | 55.2 ± 0.7 | 0.126 ± 0.008 | 0.481 ± 0.016 | 0.0028 ± 0.0010 |
| Sigma Aldrich Magnesium Hydroxide | | 20 | 1.33 ± 0.03 | 0.3648 ± 0.0005 | 0.2319 ± 0.0003 | 212 ± 3 | 33.9 ± 0.4 | 0.196 ± 0.006 | 0.447 ± 0.003 | 0.013 ± 0.003 |
| Sigma Aldrich Magnesium Hydroxide | | 20 | 1.45 ± 0.03 | 0.3637 ± 0.0003 | 0.231 ± 0.0002 | 286 ± 5 | 45.6 ± 0.8 | 0.113 ± 0.009 | 1.96 ± 0.26 | 0.009 ± 0.003 |
| CMgS | | 20 | 1.14 ± 0.03 | 0.1206 ± 0.0003 | 0.059797 ± 0.00015 | 336 ± 5 | 53.7 ± 0.8 | 0.149 ± 0.003 | 0.987 ± 0.005 | 0.0496 ± 0.010 |
| CMgS | | 20 | 1.33 ± 0.03 | 0.0805 ± 0.0003 | 0.03804 ± 0.00015 | 226 ± 3 | 36.1 ± 0.5 | 0.156 ± 0.003 | 1.089 ± 0.004 | 0.099 ± 0.03 |
| US Research Nanomaterials Inc. Magnesium Hydroxide 10 nm | | 20 | 0.75 ± 0.03 | 0.2644 ± 0.0006 | 0.1529 ± 0.0004 | 360 ± 20 | 57 ± 3 | 0.111 ± 0.008 | 1.009 ± 0.016 | 0.0075 ± 0.0006 |
| US Research Nanomaterials Inc. Magnesium Hydroxide 10 nm | | 20 | 1.45 ± 0.03 | 0.1978 ± 0.0005 | 0.1068 ± 0.0003 | 379 ± 3 | 60.5 ± 0.4 | 0.0845 ± 0.0008 | 0.559 ± 0.003 | 0.0009 ± 0.0006 |
| US Research Nanomaterials Inc. Magnesium Hydroxide 10 nm | | 20 | 0.997 ± 0.03 | 0.19154 ± 0.00099 | 0.1027 ± 0.0005 | 232 ± 3 | 37.1 ± 0.5 | 0.119 ± 0.003 | 0.89 ± 0.14 | 0.008 ± 0.002 |
| US Research Nanomaterials Inc. Magnesium Hydroxide 10 nm | | 20 | 1.51 ± 0.03 | 0.2023 ± 0.0005 | 0.1098 ± 0.0002 | 437 ± 3 | 69.7 ± 0.6 | 0.0923 ± 0.0009 | 0.602 ± 0.012 | 0.0009 ± 0.0006 |
| US Research Nanomaterials Inc. Magnesium Hydroxide 10 nm | | 20 | 0.75 ± 0.03 | 0.2656 ± 0.0005 | 0.1537 ± 0.0003 | 432 ± 15 | 34 ± 2 | 0.138 ± 0.01004 | 1.69 ± 0.02 | 0.046 ± 0.007 |
| US Research Nanomaterials Inc. Magnesium Hydroxide 10 nm | | 20 | 1.45 ± 0.03 | 0.2077 ± 0.0003 | 0.11331 ± 0.00016 | 451.8 ± 1.4 | 36.1 ± 0.2 | 0.0894 ± 0.0007 | 0.5605 ± 0.006 | 0.0049 ± 0.0011 |
| US Research Nanomaterials Inc. Magnesium Hydroxide 10 nm | | 20 | 0.997 ± 0.03 | 0.1947 ± 0.0002 | 0.104799 ± 0.00013 | 277.8 ± 1.98 | 22.18 ± 0.14 | 0.1278 ± 0.0013 | 0.796 ± 0.013 | 0.0029 ± 0.0003 |
| US Research Nanomaterials Inc. Magnesium Hydroxide 10 nm | | 20 | 1.51 ± 0.03 | 0.15123 ± 0.00017 | 0.07762 ± 0.00009 | 543 ± 4 | 43.3 ± 0.3 | 0.1045 ± 0.0011 | 1.096 ± 0.02 | 22 ± 4 |
| US Research Nanomaterials Inc. Magnesium Hydroxide 10 nm | | 20 | 0.75 ± 0.03 | 0.2592 ± 0.0005 | 0.1491 ± 0.0003 | 520 ± 40 | 20.9 ± 1.4 | 0.084 ± 0.008 | 0.746 ± 0.009 | 8 ± 6 |
| US Research Nanomaterials Inc. Magnesium Hydroxide 10 nm | | 20 | 2.22 ± 0.03 | 0.1831 ± 0.0003 | 0.097299 ± 0.00015 | 562 ± 4 | 22.4 ± 0.3 | 0.1136 ± 0.0019 | 1.071 ± 0.013 | 0.30 ± 0.13 |
| US Research Nanomaterials Inc. Magnesium Hydroxide 10 nm | | 20 | 1.16 ± 0.03 | 0.1887 ± 0.0002 | 0.10091 ± 0.00013 | 342 ± 2 | 13.7 ± 0.3 | 0.1299 ± 0.006 | 0.561 ± 0.013 | 0.00167 ± 0.00010 |
| US Research Nanomaterials Inc. Magnesium Hydroxide 10 nm | | 20 | 1.51 ± 0.03 | 0.15052 ± 0.00019 | 0.07719 ± 0.00009 | 676 ± 4 | 26.9 ± 0.3 | 0.0795 ± 0.0014 | 0.809 ± 0.004 | 12 ± 8 |
| US Research Nanomaterials Inc. Magnesium Hydroxide 10 nm | | 20 | 0.75 ± 0.03 | 0.2663 ± 0.0004 | 0.1542 ± 0.0003 | 640 ± 50 | 13 ± 4 | 0.1001 ± 0.03 | 1.09 ± 0.17 | 5.4 ± 0.4 |
| US Research Nanomaterials Inc. Magnesium Hydroxide 10 nm | | 20 | 1.45 ± 0.03 | 0.1819 ± 0.0003 | 0.09659 ± 0.00015 | 682 ± 5 | 13.6 ± 0.3 | 0.0759 ± 0.002 | 0.602 ± 0.014 | 8.3 ± 0.7 |
| US Research Nanomaterials Inc. Magnesium Hydroxide 10 nm | | 20 | 0.997 ± 0.03 | 0.1952 ± 0.0002 | 0.10509 ± 0.00013 | 413 ± 3 | 8.2 ± 0.3 | 0.1124 ± 0.005 | 0.501 ± 0.011 | 0.00225 ± 0.00199 |
| US Research Nanomaterials Inc. Magnesium Hydroxide 10 nm | | 20 | 1.51 ± 0.03 | 0.14587 ± 0.00017 | 0.07442 ± 0.00009 | 811 ± 5 | 16.2 ± 0.3 | 0.0819999 ± 0.0017 | 2.656 ± 0.006 | 14 ± 6 |
| US Research Nanomaterials Inc. Magnesium Hydroxide 10 nm | | 20 | 1.85 ± 0.03 | 0.2164 ± 0.0003 | 0.11917 ± 0.00015 | 287 ± 3.5 | 45.9 ± 0.6 | 0.0689 ± 0.0011 | 0.742 ± 0.005 | 0.005 ± 0.003 |
| US Research Nanomaterials Inc. Magnesium Hydroxide 10 nm | | 20 | 2.05 ± 0.03 | 0.2149 ± 0.0003 | 0.11819 ± 0.00015 | 228 ± 2.7 | 36.4 ± 0.6 | 0.0743 ± 0.0013 | 0.6997 ± 0.006 | 0.00243 ± 0.00015 |
| US Research Nanomaterials Inc. Magnesium Hydroxide 10 nm | | 20 | 1.07 ± 0.03 | 0.2284 ± 0.0003 | 0.12731 ± 0.00014 | 262 ± 3 | 41.8 ± 0.6 | 0.189 ± 0.017 | 0.59 ± 0.06 | 0.0039 ± 0.0015 |
| US Research Nanomaterials Inc. Magnesium Hydroxide 10 nm | | 20 | 1.17 ± 0.03 | 0.1798 ± 0.002 | 0.0952 ± 0.0011 | 236 ± 6 | 37.7 ± 1.4 | 0.0164 ± 0.0008 | 0.69 ± 0.05 | 0.0027 ± 0.0004 |
| US Research Nanomaterials Inc. Magnesium Hydroxide 10 nm | | 20 | 1.85 ± 0.03 | 0.225 ± 0.0003 | 0.12502 ± 0.00016 | 343 ± 4 | 27.3 ± 0.6 | 0.062 ± 0.006 | 0.95 ± 0.06 | 0.0029 ± 0.0016 |
| US Research Nanomaterials Inc. Magnesium Hydroxide 10 nm | | 20 | 2.05 ± 0.03 | 0.2352 ± 0.0002 | 0.13201 ± 0.00013 | 266 ± 3 | 21.3 ± 0.6 | 0.074 ± 0.004 | 0.732 ± 0.018 | 0.005 ± 0.003 |
| US Research Nanomaterials Inc. Magnesium Hydroxide 10 nm | | 20 | 1.07 ± 0.03 | 0.2397 ± 0.0002 | 0.13522 ± 0.00014 | 309.6 ± 4 | 24.7 ± 0.6 | 0.121 ± 0.003 | 0.532 ± 0.004 | 0.003 ± 0.002 |
| US Research Nanomaterials Inc. Magnesium Hydroxide 10 nm | | 20 | 1.17 ± 0.03 | 0.1893 ± 0.0002 | 0.1012 ± 0.0011 | 278 ± 6.5 | 22.2 ± 0.9 | 0.4 ± 0.3 | 0.47 ± 0.17 | 0.002 ± 0.002 |
| US Research Nanomaterials Inc. Magnesium Hydroxide 10 nm | | 20 | 1.85 ± 0.03 | 0.2264 ± 0.0003 | 0.12599 ± 0.00016 | 408 ± 5 | 16.3 ± 0.6 | 0.0668 ± 0.003 | 0.63 ± 0.04 | 0.00151 ± 0.00017 |
| US Research Nanomaterials Inc. Magnesium Hydroxide 10 nm | | 20 | 2.05 ± 0.03 | 0.2361 ± 0.0002 | 0.13267 ± 0.00013 | 318 ± 4 | 12.7 ± 0.6 | 0.0694 ± 0.004 | 0.617 ± 0.0097 | 0.00151 ± 0.00015 |
| US Research Nanomaterials Inc. Magnesium Hydroxide 10 nm | | 20 | 1.07 ± 0.03 | 0.2414 ± 0.0002 | 0.13637 ± 0.00014 | 367 ± 4.5 | 14.7 ± 0.6 | 0.1201 ± 0.005 | 0.555 ± 0.003 | 0.0028 ± 0.0004 |
| US Research Nanomaterials Inc. Magnesium Hydroxide 10 nm | | 20 | 1.17 ± 0.03 | 0.193 ± 0.002 | 0.104 ± 0.011 | 331 ± 8.5 | 13.2 ± 1.4 | 0.0314 ± 0.004 | 1.02 ± 0.07 | 0.0020 ± 0.0004 |
| CMgS | | 30 | 1.39 ± 0.03 | 0.1395 ± 0.0004 | 0.0707 ± 0.0002 | 392 ± 5 | 62.6 ± 0.8 | 0.0964 ± 0.0012 | 1.951 ± 0.013 | 19.4 ± 0.6 |
| CMgS | | 30 | 1.39 ± 0.03 | 0.1398 ± 0.0007 | 0.0708 ± 0.0004 | 392 ± 6 | 62.6 ± 0.9 | 0.0968 ± 0.0014 | 2.215 ± 0.014 | 20.2 ± 0.6 |
| CMgS | | 30 | 1.39 ± 0.03 | 0.1435 ± 0.0005 | 0.07299 ± 0.0002 | 391 ± 5 | 62.4 ± 0.8 | 0.0887 ± 0.0011 | 1.734 ± 0.009 | 0.0080 ± 0.0003 |
| CMgS | | 30 | 1.39 ± 0.03 | 0.1522 ± 0.0005 | 0.0782 ± 0.0002 | 389 ± 5 | 62.2 ± 0.8 | 0.0817 ± 0.00105 | 1.613 ± 0.014 | 0.0077 ± 0.0003 |
| CMgS | | 30 | 1.39 ± 0.03 | 0.1628 ± 0.0005 | 0.0847 ± 0.0003 | 388 ± 5 | 61.9 ± 0.8 | 0.0538 ± 0.0007 | 0.516 ± 0.003 | 0.0016 ± 0.0015 |
| CMgS | | 30 | 1.39 ± 0.03 | 0.1655 ± 0.0006 | 0.0863 ± 0.0003 | 387 ± 5 | 61.7 ± 0.8 | 0.0495 ± 0.0007 | 0.729 ± 0.004 | 0.0037 ± 0.0015 |
| CMgS | | 30 | 1.12 ± 0.03 | 0.127077 ± 0.00045 | 0.06346 ± 0.0002 | 392 ± 5 | 62.6 ± 0.8 | 0.051938676 ± 0.00065 | 1.027 ± 0.002 | 0.26 ± 0.11 |
| CMgS | | 30 | 1.12 ± 0.03 | 0.13502 ± 0.0005 | 0.06804 ± 0.0002 | 390.3 ± 5 | 62.3 ± 0.8 | 0.046460045 ± 0.0006 | 1.0185 ± 0.0015 | 0.11 ± 0.11 |
| CMgS | | 30 | 1.12 ± 0.03 | 0.14479 ± 0.00055 | 0.07377 ± 0.0003 | 389 ± 5 | 62.1 ± 0.8 | 0.043045834 ± 0.0006 | 0.812 ± 0.003 | 0.0016 ± 0.0015 |
| CMgS | | 30 | 1.12 ± 0.03 | 0.1492 ± 0.0006 | 0.0764 ± 0.0003 | 388 ± 5 | 61.9 ± 0.8 | 0.0408 ± 0.0006 | 0.809 ± 0.003 | 0.0041 ± 0.0015 |
| CMgS | | 30 | 1.12 ± 0.03 | 0.1575 ± 0.0005 | 0.08138 ± 0.00025 | 387 ± 5 | 61.8 ± 0.8 | 0.0382 ± 0.0005 | 0.677 ± 0.003 | 0.0013 ± 0.0015 |
| CMgS | | 30 | 1.12 ± 0.03 | 0.16707 ± 0.0005 | 0.08726 ± 0.0003 | 385.6 ± 5 | 61.5 ± 0.8 | 0.0361 ± 0.0005 | 0.6185 ± 0.002 | 0.0046 ± 0.0015 |
| CMgS | | 30 | 0.99 ± 0.03 | 0.1468 ± 0.0006 | 0.07497 ± 0.0003 | 408 ± 5 | 65.2 ± 0.9 | 0.0644 ± 0.0008 | 1.226 ± 0.007 | 0.15 ± 0.07 |
| CMgS | | 30 | 0.99 ± 0.03 | 0.14896 ± 0.0006 | 0.0763 ± 0.0003 | 402 ± 5 | 64.2 ± 0.9 | 0.0649 ± 0.0009 | 1.122 ± 0.007 | 0.04 ± 0.07 |
| CMgS | | 30 | 0.99 ± 0.03 | 0.1552 ± 0.0005 | 0.08003 ± 0.0003 | 407 ± 5 | 65 ± 0.8 | 0.0675 ± 0.0009 | 1.244 ± 0.006 | 0.0100 ± 0.0005 |
| CMgS | | 30 | 0.99 ± 0.03 | 0.168899 ± 0.0005 | 0.0884 ± 0.0003 | 405 ± 5 | 64.6 ± 0.8 | 0.07499 ± 0.0009 | 1.663 ± 0.005 | 0.0094 ± 0.0005 |
| CMgS | | 30 | 0.99 ± 0.03 | 0.17976 ± 0.0006 | 0.0952 ± 0.0003 | 403 ± 5 | 64.4 ± 0.8 | 0.0732 ± 0.00096 | 1.529 ± 0.0085 | 0.0060 ± 0.0012 |
| CMgS | | 30 | 0.99 ± 0.03 | 0.1835 ± 0.0008 | 0.0976 ± 0.0004 | 403 ± 6 | 64.3 ± 0.9 | 0.07143 ± 0.0010 | 1.323 ± 0.007 | 0.0077 ± 0.0012 |
| CMgS | | 30 | 1.17 ± 0.03 | 0.1487 ± 0.0005 | 0.0761 ± 0.0002 | 405.74 ± 5 | 64.8 ± 0.8 | 0.05527 ± 0.0007 | 0.7224 ± 0.002 | 0.07 ± 0.02 |
| CMgS | | 30 | 1.17 ± 0.03 | 0.1543 ± 0.0005 | 0.0795 ± 0.0002 | 405.28 ± 5 | 64.7 ± 0.8 | 0.052888 ± 0.0007 | 0.7251 ± 0.003 | 0.04 ± 0.02 |
| CMgS | | 30 | 1.17 ± 0.03 | 0.1566 ± 0.0006 | 0.0808 ± 0.0003 | 404.92 ± 5 | 64.6 ± 0.9 | 0.05201 ± 0.0007 | 0.7146 ± 0.003 | 0.00454 ± 0.000017 |
| CMgS | | 30 | 1.17 ± 0.03 | 0.1578 ± 0.0006 | 0.0816 ± 0.0003 | 404.69 ± 5 | 64.6 ± 0.9 | 0.0498 ± 0.0007 | 0.7364 ± 0.003 | 0.00457 ± 0.000017 |
| CMgS | | 30 | 1.17 ± 0.03 | 0.16197 ± 0.0005 | 0.0841 ± 0.0003 | 404.06 ± 5 | 64.5 ± 0.8 | 0.0464 ± 0.0006 | 0.6592 ± 0.003 | 0.0025 ± 0.0003 |
| CMgS | | 30 | 1.17 ± 0.03 | 0.16815 ± 0.0005 | 0.0879 ± 0.00025 | 403.58 ± 5 | 64.4 ± 0.8 | 0.0486 ± 0.0006 | 0.8308 ± 0.003 | 0.0021 ± 0.0003 |
| US Research Nanomaterials Inc. Magnesium Hydroxide 10 nm | | 60 | 1.38 ± 0.03 | 0.3021 ± 0.0012 | 0.1812 ± 0.0007 | 294 ± 8 | 46.9 ± 1.4 | 0.0468 ± 0.0013 | 1.35 ± 0.07 | 0.002 ± 0.002 |
| US Research Nanomaterials Inc. Magnesium Hydroxide 10 nm | | 60 | 1.40 ± 0.03 | 0.3073 ± 0.0013 | 0.1852 ± 0.0002 | 297 ± 8 | 47.4 ± 1.4 | 0.0272 ± 0.0008 | 0.5704 ± 0.003 | 0.008 ± 0.011 |
| US Research Nanomaterials Inc. Magnesium Hydroxide 10 nm | | 60 | 1.00 ± 0.03 | 0.2757 ± 0.0013 | 0.1612 ± 0.0008 | 301 ± 9 | 48.04 ± 1.4 | 0.04399 ± 0.0013 | 0.63 ± 0.03 | 0.024 ± 0.003 |
| US Research Nanomaterials Inc. Magnesium Hydroxide 10 nm | | 60 | 1.18 ± 0.03 | 0.1939 ± 0.0013 | 0.1043 ± 0.0007 | 305 ± 9.5 | 48.8 ± 1.4 | 0.05697 ± 0.0018 | 1.07 ± 0.04 | 0.0869 ± 0.00097 |
| US Research Nanomaterials Inc. Magnesium Hydroxide 10 nm | | 60 | 1.22 ± 0.03 | 0.29403 ± 0.0016 | 0.17499 ± 0.00097 | 295 ± 9 | 46.9 ± 1.4 | 0.0439 ± 0.0014 | 1.611 ± 0.004 | 0.08 ± 0.05 |
| US Research Nanomaterials Inc. Magnesium Hydroxide 10 nm | | 60 | 1.26 ± 0.03 | 0.2924 ± 0.0011 | 0.1738 ± 0.0007 | 298 ± 8 | 47.5 ± 1.4 | 0.0214 ± 0.0006 | 0.682 ± 0.008 | 0.02 ± 0.02 |
| US Research Nanomaterials Inc. Magnesium Hydroxide 10 nm | | 60 | 1.02 ± 0.03 | 0.2622 ± 0.0015 | 0.1513 ± 0.0009 | 303 ± 9.99 | 48.4 ± 1.4 | 0.0441 ± 0.0014 | 0.671 ± 0.00998 | 0.04 ± 0.04 |
| US Research Nanomaterials Inc. Magnesium Hydroxide 10 nm | | 60 | 1.17 ± 0.03 | 0.19777 ± 0.00102 | 0.1067 ± 0.0005 | 305 ± 9 | 48.4 ± 1.4 | 0.0505 ± 0.0015 | 0.87 ± 0.02 | 0.0181 ± 0.0004 |
| US Research Nanomaterials Inc. Magnesium Hydroxide 10 nm | | 50 | 0.93 ± 0.03 | 0.2841 ± 0.0011 | 0.1674 ± 0.0006 | 308 ± 5.5 | 49.1 ± 0.9 | 0.0543 ± 0.0012 | 0.68 ± 0.04 | 0.0024 ± 0.0012 |
| US Research Nanomaterials Inc. Magnesium Hydroxide 10 nm | | 50 | 1.01 ± 0.03 | 0.2581 ± 0.0009 | 0.1483 ± 0.0005 | 311 ± 5 | 49.6 ± 0.9 | 0.0498 ± 0.0018 | 0.905 ± 0.05 | 1.2 ± 0.8 |
| US Research Nanomaterials Inc. Magnesium Hydroxide 10 nm | | 50 | 0.97 ± 0.03 | 0.24144 ± 0.00105 | 0.1364 ± 0.0006 | 315 ± 6 | 50.2 ± 0.9 | 0.0829 ± 0.0017 | 0.857 ± 0.006 | 25 ± 3 |
| US Research Nanomaterials Inc. Magnesium Hydroxide 10 nm | | 50 | 1.16 ± 0.03 | 0.2882 ± 0.0009 | 0.1705 ± 0.0005 | 314 ± 5 | 50.1 ± 0.9 | 0.0434 ± 0.0011 | 0.693 ± 0.008 | 0.025 ± 0.011 |
| CMgS | | 40 | 1.04 ± 0.03 | 0.0345 ± 0.0006 | 0.0154 ± 0.0003 | 343 ± 8 | 54.6 ± 1.4 | 0.0439 ± 0.0013 | 0.742 ± 0.011 | 0.003 ± 0.004 |
| CMgS | | 40 | 1.03 ± 0.03 | 0.01205 ± 0.0005 | 0.0052 ± 0.0002 | 342 ± 16 | 54.6 ± 2 | 0.055 ± 0.002 | 1.95 ± 0.19 | 0.2 ± 0.2 |
| CMgS | | 40 | 0.97 ± 0.03 | 0.0684 ± 0.0006 | 0.0318 ± 0.0003 | 345 ± 7 | 55.1 ± 0.9 | 0.0519 ± 0.0010 | 0.636 ± 0.017 | 0.012 ± 0.002 |
| CMgS | | 40 | 1.16 ± 0.03 | 0.0501 ± 0.0006 | 0.0228 ± 0.0003 | 345 ± 7 | 55.1 ± 0.9 | 0.0558 ± 0.0012 | 1.104 ± 0.02 | 0.05 ± 0.03 |
| US Research Nanomaterials Inc. Magnesium Hydroxide 10 nm | | 50 | 0.92 ± 0.03 | 0.202 ± 0.003 | 0.1095 ± 0.0014 | 320 ± 14 | 51.2 ± 2 | 0.058 ± 0.003 | 1.32 ± 0.03 | 0.016 ± 0.003 |
| US Research Nanomaterials Inc. Magnesium Hydroxide 10 nm | | 50 | 0.98 ± 0.03 | 0.192 ± 0.002 | 0.1028 ± 0.0012 | 320 ± 12 | 51.2 ± 1.9 | 0.042 ± 0.0016 | 1.5899 ± 0.009 | 17 ± 7 |
| US Research Nanomaterials Inc. Magnesium Hydroxide 10 nm | | 50 | 0.99 ± 0.03 | 0.247 ± 0.002 | 0.1403 ± 0.0013 | 312.7 ± 10.5 | 49.8 ± 1.4 | 0.0457 ± 0.0015 | 0.533 ± 0.004 | 0.017 ± 0.004 |
| US Research Nanomaterials Inc. Magnesium Hydroxide 10 nm | | 50 | 1.18 ± 0.03 | 0.2413 ± 0.0017 | 0.1363 ± 0.0009 | 322 ± 9 | 51.2 ± 1.4 | 0.0561 ± 0.0015 | 1.113 ± 0.007 | 0.0497 ± 0.0198 |
| US Research Nanomaterials Inc. Magnesium Hydroxide 10 nm | | 60 | 0.74 ± 0.03 | 0.2011 ± 0.0016 | 0.10901 ± 0.0009 | 308 ± 11 | 49.3 ± 1.9 | 0.0501 ± 0.005 | 0.86 ± 0.04 | 0.009 ± 0.007 |
| US Research Nanomaterials Inc. Magnesium Hydroxide 10 nm | | 60 | 1.11 ± 0.03 | 0.226 ± 0.002 | 0.1255 ± 0.0012 | 307 ± 13 | 48.8 ± 1.9 | 0.035 ± 0.007 | 0.93 ± 0.03 | 0.00287 ± 0.00015 |
| US Research Nanomaterials Inc. Magnesium Hydroxide 10 nm | | 60 | 0.70 ± 0.03 | 0.1979 ± 0.0009 | 0.1068 ± 0.0005 | 312 ± 9 | 49.8 ± 1.4 | 0.058 ± 0.003 | 0.864 ± 0.004 | 0.0022 ± 0.0005 |
| US Research Nanomaterials Inc. Magnesium Hydroxide 10 nm | | 60 | 0.96 ± 0.03 | 0.152 ± 0.002 | 0.0779 ± 0.0011 | 311 ± 8 | 49.8 ± 1.4 | 0.043 ± 0.005 | 1.18 ± 0.17 | 0.0036 ± 0.0008 |
| CMgS | | 30 | 0.93 ± 0.03 | 0.1329 ± 0.0016 | 0.0668 ± 0.0008 | 395 ± 7 | 63.2 ± 0.9 | 0.0503 ± 0.0014 | 0.595 ± 0.004 | 0.014 ± 0.002 |
| CMgS | | 30 | 1.30 ± 0.03 | 0.1505 ± 0.0018 | 0.0772 ± 0.0009 | 390 ± 9.6 | 62.4 ± 1.4 | 0.0289 ± 0.0008 | 0.689 ± 0.005 | 0.0107 ± 0.0007 |
| CMgS | | 30 | 1.08 ± 0.03 | 0.1551 ± 0.0015 | 0.0799 ± 0.0008 | 406 ± 8 | 64.7 ± 1.4 | 0.0462 ± 0.0013 | 0.578 ± 0.009 | 0.0083 ± 0.0013 |
| CMgS | | 30 | 1.09 ± 0.03 | 0.1817 ± 0.0012 | 0.0964 ± 0.0006 | 403 ± 7 | 64.2 ± 0.9 | 0.0441 ± 0.0011 | 0.601 ± 0.002 | 0.00257 ± 0.00009 |
| Sigma Aldrich Alumium Oxide <100 nm | | 20 | 1.14 ± 0.03 | 0.11071 ± 0.00012 | 0.054247 ± 0.0006 | 498 ± 6 | 55.2 ± 0.7 | 0.403 ± 0.012 | 1.23 ± 0.04 | 0.056 ± 0.007 |
| Sigma Aldrich Alumium Oxide <100 nm | | 20 | 1.33 ± 0.03 | 0.1324 ± 0.0007 | 0.0665 ± 0.0003 | 396 ± 9 | 78 ± 4 | 0.55 ± 0.03 | 1.369 ± 0.019 | 0.09 ± 0.02 |
| Sigma Aldrich Alumium Oxide <100 nm | | 20 | 1.14 ± 0.03 | 0.2025 ± 0.0003 | 0.10989 ± 0.00017 | 451 ± 5 | 69 ± 3 | 0.222 ± 0.011 | 0.62 ± 0.03 | 0.00181 ± 0.00008 |
| Sigma Aldrich Alumium Oxide <100 nm | | 20 | 1.33 ± 0.03 | 0.2409 ± 0.0014 | 0.13605 ± 0.0008 | 369 ± 6 | 73 ± 3 | 0.387 ± 0.0199 | 0.81 ± 0.03 | 0.0026 ± 0.0006 |
| Sigma Aldrich Alumium Oxide <100 nm | | 30 | 0.98 ± 0.03 | 0.0696 ± 0.0002 | 0.02125 ± 0.00007 | 431 ± 5 | 68.8 ± 0.7 | 0.2002 ± 0.018 | 2.2 ± 0.4 | 1.4 ± 0.6 |
| Sigma Aldrich Alumium Oxide <100 nm | | 30 | 1.09 ± 0.03 | 0.06805 ± 0.0003 | 0.02068 ± 0.00009 | 325 ± 4 | 51.8 ± 0.7 | 0.173 ± 0.004 | 2.34 ± 0.07 | 18 ± 11 |
| Sigma Aldrich Alumium Oxide <100 nm | | 30 | 1.04 ± 0.03 | 0.0608 ± 0.0004 | 0.01815 ± 0.00012 | 319 ± 5 | 50.9 ± 0.8 | 0.181 ± 0.003 | 1.85 ± 0.02 | 24 ± 17 |
| Sigma Aldrich Alumium Oxide <100 nm | | 60 | 1.16 ± 0.03 | 0.1792 ± 0.0007 | 0.0694 ± 0.0003 | 331 ± 9 | 52.8 ± 1.4 | 0.079 ± 0.002 | 1.009 ± 0.017 | 0.038 ± 0.014 |
| Sigma Aldrich Alumium Oxide <100 nm | | 60 | 1.09 ± 0.03 | 0.1699 ± 0.0006 | 0.0646 ± 0.0002 | 325 ± 8 | 51.7 ± 1.4 | 0.0729 ± 0.0018 | 0.8499 ± 0.011 | 0.060 ± 0.011 |
| Sigma Aldrich Alumium Oxide <100 nm | | 60 | 0.85 ± 0.03 | 0.1313 ± 0.0006 | 0.0461 ± 0.0002 | 318 ± 9 | 50.8 ± 1.4 | 0.0704 ± 0.0019 | 0.698 ± 0.009 | 0.036 ± 0.006 |
| Sigma Aldrich Alumium Oxide <100 nm | | 60 | 0.79 ± 0.03 | 0.1125 ± 0.0006 | 0.0379 ± 0.0002 | 310.5 ± 10.2 | 49.3 ± 1.4 | 0.091 ± 0.003 | 0.91 ± 0.02 | 0.105 ± 0.003 |
| Sigma Aldrich Alumium Oxide <100 nm | | 60 | 0.997 ± 0.03 | 0.0684 ± 0.0007 | 0.0208 ± 0.0002 | 321 ± 11 | 51.2 ± 1.9 | 0.0669 ± 0.0009 | 0.7166 ± 0.039 | 0.012 ± 0.003 |
| Sigma Aldrich Alumium Oxide <100 nm | | 60 | 1.14 ± 0.03 | 0.0609 ± 0.0005 | 0.01819 ± 0.00015 | 316 ± 9 | 50.3 ± 1.4 | 0.0728 ± 0.0005 | 0.792 ± 0.024 | 0.04 ± 0.04 |
| Sigma Aldrich Alumium Oxide <100 nm | | 60 | 1.02 ± 0.03 | 0.0316 ± 0.0007 | 0.00875 ± 0.00019 | 313 ± 15 | 50 ± 2 | 0.07699 ± 0.0007 | 0.955 ± 0.027 | 0.05 ± 0.05 |
| Sigma Aldrich Alumium Oxide <100 nm | | 60 | 0.85 ± 0.03 | 0.0074 ± 0.0007 | 0.00193 ± 0.00017 | 303 ± 30 | 48 ± 5 | 0.0888 ± 0.0012 | 0.87 ± 0.05 | 0.04 ± 0.04 |
| Sigma Aldrich Alumium Oxide <100 nm | | 50 | 0.94 ± 0.03 | 0.0673 ± 0.004 | 0.0204 ± 0.0013 | 347 ± 5 | 55.4 ± 0.9 | 0.172 ± 0.006 | 2.29 ± 0.18 | 41 ± 5 |
| Sigma Aldrich Alumium Oxide <100 nm | | 50 | 1.03 ± 0.03 | 0.1048 ± 0.0008 | 0.0347 ± 0.0003 | 330 ± 8 | 52.7 ± 1.4 | 0.195 ± 0.005 | 3.305 ± 0.098 | 29.6 ± 1.9 |
| Sigma Aldrich Alumium Oxide <100 nm | | 50 | 0.82 ± 0.03 | 0.048 ± 0.003 | 0.0138 ± 0.0008 | 340 ± 40 | 54 ± 7 | 0.15 ± 0.02 | 2.5 ± 0.7 | 33 ± 4 |
| Sigma Aldrich Alumium Oxide <100 nm | | 40 | 1.14 ± 0.03 | 0.0205 ± 0.00025 | 0.005514 ± 0.00007 | 356 ± 8 | 57 ± 0.9 | 0.189 ± 0.004 | 2.83 ± 0.12 | 0.86 ± 0.11 |
| Sigma Aldrich Alumium Oxide <100 nm | | 40 | 1.08 ± 0.03 | 0.02275 ± 0.0003 | 0.00615 ± 0.00009 | 336 ± 8 | 53.6 ± 1.4 | 0.189 ± 0.005 | 2.81 ± 0.12 | 11 ± 2 |
| Sigma Aldrich Alumium Oxide <100 nm | | 40 | 1.04 ± 0.03 | 0.0076 ± 0.0003 | 0.00196 ± 0.00008 | 325 ± 15 | 51.7 ± 2 | 0.2319 ± 0.0105 | 4.1 ± 0.5 | 7 ± 4 |
| Sigma Aldrich Alumium Oxide <100 nm | | 40 | 0.80 ± 0.03 | 0.0216 ± 0.0004 | 0.00582 ± 0.00011 | 324 ± 9 | 51.7 ± 1.4 | 0.646 ± 0.016 | 24 ± 3 | 12.4 ± 0.5 |
| Sigma Aldrich Alumium Oxide <100 nm | | 50 | 1.14 ± 0.03 | 0.0999 ± 0.0007 | 0.0327 ± 0.0002 | 339 ± 8 | 54.1 ± 1.4 | 0.167 ± 0.004 | 2.924 ± 0.018 | 3.8 ± 1.4 |
| Sigma Aldrich Alumium Oxide <100 nm | | 50 | 1.08 ± 0.03 | 0.1024 ± 0.0006 | 0.0338 ± 0.0002 | 335 ± 7 | 53.6 ± 0.9 | 0.119 ± 0.003 | 1.559 ± 0.014 | 4 ± 2 |
| Sigma Aldrich Alumium Oxide <100 nm | | 50 | 1.02 ± 0.03 | 0.0961 ± 0.0008 | 0.0312 ± 0.0003 | 332 ± 9 | 52.9 ± 1.4 | 0.141 ± 0.004 | 2.27 ± 0.04 | 7 ± 4 |
| Sigma Aldrich Alumium Oxide <100 nm | | 50 | 0.81 ± 0.03 | 0.1068 ± 0.0007 | 0.0356 ± 0.0002 | 322 ± 7 | 51.2 ± 0.9 | 0.201 ± 0.004 | 3.14 ± 0.06 | 6.6 ± 0.8 |
| Sigma Aldrich Alumium Oxide <100 nm | | 60 | 0.93 ± 0.03 | 0.2081 ± 0.0005 | 0.085 ± 0.0002 | 328 ± 8 | 52.2 ± 1.4 | 0.093 ± 0.004 | 1.12 ± 0.04 | 0.011 ± 0.006 |
| Sigma Aldrich Alumium Oxide <100 nm | | 60 | 0.87 ± 0.03 | 0.1818 ± 0.0005 | 0.07074 ± 0.00019 | 329 ± 8 | 52.2 ± 0.9 | 0.092 ± 0.005 | 1.21 ± 0.06 | 0.03 ± 0.02 |
| Sigma Aldrich Alumium Oxide <100 nm | | 60 | 0.82 ± 0.03 | 0.1507 ± 0.0006 | 0.0551 ± 0.0002 | 320 ± 40 | 51 ± 7 | 0.126 ± 0.017 | 2.8 ± 0.6 | 7 ± 7 |
| Sigma Aldrich Alumium Oxide <100 nm | | 60 | 0.60 ± 0.03 | 0.1304 ± 0.0019 | 0.0457 ± 0.0006 | 312 ± 17 | 50 ± 3 | 0.125 ± 0.02 | 1.9 ± 0.2 | 5 ± 4 |
| Sigma Aldrich Alumium Oxide <100 nm | | 30 | 1.14 ± 0.03 | 0.0672 ± 0.0004 | 0.02037 ± 0.00012 | 435 ± 6 | 69.4 ± 0.9 | 0.133 ± 0.004 | 1.46 ± 0.02 | 0.14 ± 0.06 |
| Sigma Aldrich Alumium Oxide <100 nm | | 30 | 1.08 ± 0.03 | 0.065155 ± 0.0003 | 0.019665 ± 0.000098 | 418 ± 6 | 66.8 ± 0.7 | 0.122 ± 0.002 | 1.408 ± 0.0099 | 2.1 ± 1.9 |
| Sigma Aldrich Alumium Oxide <100 nm | | 30 | 1.11 ± 0.03 | 0.06448 ± 0.0004 | 0.01943 ± 0.00012 | 400 ± 6 | 63.7 ± 0.9 | 0.178 ± 0.003 | 2.94 ± 0.03 | 5 ± 5 |
| Sigma Aldrich Alumium Oxide <100 nm | | 30 | 0.84 ± 0.03 | 0.0754 ± 0.0003 | 0.02333 ± 0.00011 | 391 ± 5 | 62.5 ± 0.9 | 0.177 ± 0.004 | 1.92 ± 0.04 | 12 ± 2 |
| Sigma Aldrich Alumium Oxide <100 nm | | 20 | 1.64 ± 0.03 | 0.09707 ± 0.00008 | 0.03161 ± 0.00003 | 541 ± 3.5 | 86.4 ± 0.6 | 0.232 ± 0.005 | 1.019 ± 0.014 | 0.00997 ± 0.0013 |
| Sigma Aldrich Alumium Oxide <100 nm | | 20 | 1.33 ± 0.03 | 0.10748 ± 0.00007 | 0.03584 ± 0.00002 | 822 ± 6 | 131.2 ± 0.9 | 0.1046 ± 0.0009 | 0.925 ± 0.005 | 0.06 ± 0.02 |
| Sigma Aldrich Alumium Oxide <100 nm | | 20 | 1.01 ± 0.03 | 0.10878 ± 0.00016 | 0.03638 ± 0.00005 | 498 ± 4 | 79.6 ± 0.7 | 0.266 ± 0.003 | 0.718 ± 0.007 | 0.05 ± 0.03 |
| Sigma Aldrich Alumium Oxide <100 nm | | 20 | 1.80 ± 0.03 | 0.1058 ± 0.0003 | 0.03515 ± 0.00009 | 699 ± 6.5 | 111.6 ± 0.9 | 0.1391 ± 0.0014 | 0.955 ± 0.014 | 0.00067 ± 0.00013 |
| Sigma Aldrich Alumium Oxide <100 nm | | 20 | 0.58 ± 0.03 | 0.09861 ± 0.00006 | 0.032225 ± 0.000019 | 652 ± 4 | 52.1 ± 0.3 | 0.296 ± 0.011 | 1.23 ± 0.05 | 13 ± 3 |
| Sigma Aldrich Alumium Oxide <100 nm | | 20 | 0.76 ± 0.03 | 0.10491 ± 0.00006 | 0.03478 ± 0.00002 | 985 ± 7 | 78.6 ± 0.5 | 0.184 ± 0.013 | 3.37 ± 0.3 | 22 ± 4 |
| Sigma Aldrich Alumium Oxide <100 nm | | 20 | 0.49 ± 0.03 | 0.10132 ± 0.00015 | 0.03332 ± 0.00005 | 605 ± 50 | 48.3 ± 4 | 7.5 ± 0.7 | 580 ± 30 | 17 ± 4 |
| Sigma Aldrich Alumium Oxide <100 nm | | 20 | 0.51 ± 0.03 | 0.1042 ± 0.0002 | 0.03447 ± 0.00009 | 845 ± 8 | 67.5 ± 0.6 | 0.171 ± 0.003 | 0.98 ± 0.04 | 6 ± 4 |
| Sigma Aldrich Alumium Oxide <100 nm | | 20 | 0.58 ± 0.03 | 0.08968 ± 0.00006 | 0.028712 ± 0.000019 | 791 ± 5 | 31.6 ± 0.3 | 0.2536 ± 0.003 | 0.9201 ± 0.0101 | 6 ± 5 |
| Sigma Aldrich Alumium Oxide <100 nm | | 20 | 0.76 ± 0.03 | 0.09562 ± 0.00006 | 0.031038 ± 0.000019 | 1185 ± 8 | 47.3 ± 0.3 | 0.197 ± 0.002 | 4.07 ± 0.09 | 12 ± 15 |
| Sigma Aldrich Alumium Oxide <100 nm | | 20 | 0.49 ± 0.03 | 0.09626 ± 0.00015 | 0.03129 ± 0.00005 | 728 ± 7 | 29.1 ± 0.5 | 5.4 ± 0.2 | 360 ± 30 | 9.8 ± 8 |
| Sigma Aldrich Alumium Oxide <100 nm | | 20 | 0.51 ± 0.03 | 0.102002 ± 0.0002 | 0.03359 ± 0.00008 | 1010 ± 9 | 40.3 ± 0.4 | 0.1565 ± 0.0019 | 0.9968 ± 0.0199 | 3 ± 4 |
| Sigma Aldrich Alumium Oxide <100 nm | | 20 | 0.58 ± 0.03 | 0.09102 ± 0.00005 | 0.029231 ± 0.000019 | 974 ± 7 | 19.4 ± 0.3 | 0.2669 ± 0.005 | 1.234 ± 0.019 | 8 ± 5 |
| Sigma Aldrich Alumium Oxide <100 nm | | 20 | 0.76 ± 0.03 | 0.09715 ± 0.00006 | 0.031644 ± 0.000019 | 1452 ± 9.8 | 28.9 ± 0.3 | 0.945 ± 0.012 | 140 ± 60 | 21 ± 17 |
| Sigma Aldrich Alumium Oxide <100 nm | | 20 | 0.49 ± 0.03 | 0.09798 ± 0.00014 | 0.03197 ± 0.00005 | 895 ± 80 | 18 ± 4 | 3.67 ± 0.9 | 187 ± 3 | 20 ± 19 |
| Sigma Aldrich Alumium Oxide <100 nm | | 20 | 0.51 ± 0.03 | 0.1034 ± 0.0003 | 0.03416 ± 0.00008 | 1274 ± 10.1 | 25.4 ± 0.4 | 0.1501 ± 0.003 | 1.103 ± 0.02 | 11 ± 14 |

**Table S2.** Temperature measured in the irradiated cuvette used in the bubble formation experiment. The temperature was recorded after it had increased by more than 0.2 K over the previously reported temperature. This measurement was made using a thermocouple in the irradiated region.

| Irradiation Time  s | Temperature  K |
| --- | --- |
| 1.4 | 297.55 |
| 61.99 | 297.75 |
| 87.4 | 297.95 |
| 99.8 | 298.15 |
| 113.8 | 298.35 |
| 117.4 | 298.55 |
| 129.7 | 298.75 |
| 136.4 | 298.95 |
| 141.8 | 299.15 |
| 149.8 | 299.35 |
| 155.8 | 299.55 |
| 163.8 | 299.85 |
| 173.8 | 300.05 |
| 181.4 | 300.25 |
| 187.7 | 300.45 |
| 195.8 | 300.65 |
| 201.9 | 300.85 |
| 205.7 | 301.05 |
| 211.8 | 301.25 |
| 217.8 | 301.45 |
| 223.8 | 301.65 |
| 233.7 | 301.85 |
| 239.6 | 302.05 |
| 245.5 | 302.25 |
| 249.8 | 302.45 |
| 255.8 | 302.65 |
| 259.8 | 302.85 |
| 265.8 | 303.05 |
| 271.6 | 303.25 |
| 277.8 | 303.45 |
| 283.8 | 303.65 |
| 289.5 | 303.85 |
| 295.8 | 304.05 |
| 299.9 | 304.25 |
| 305.5 | 304.45 |
| 309.7 | 304.65 |
| 317.7 | 304.85 |

VIDEOS

**Video S1.** A sequential series of x-ray transmission images covering the first 49 seconds of white beam irradiation of the sample in the region where this first bubble formed. This video shows the initial formation of this first bubble. The top left time stamp indicates the time since the start of irradiation for each x-ray image. The scale of each x-ray image is the same and indicate by the scale bar in the top left corner.

REFERENCES

(1) O’Leary, M. Irradiated Sludge Simulants, Queen’s University Belfast, 2019.

(2) Jackson, J. D. *Classical Electrodynamics*; John Wiley & Sons, 2012.

(3) Source, D. L. Diamond Machine Science http://www.diamond.ac.uk/Science/Machine.html (accessed Oct 15, 2020).

(4) Materlik, G.; Rayment, T.; Stuart, D. I. Diamond Light Source: Status and Perspectives. *Philos. Trans. R. Soc. A Math. Phys. Eng. Sci.* **2015**, *373* (2036), 20130161. https://doi.org/10.1098/rsta.2013.0161.

(5) Source, D. L. Diamond B16 Information http://www.diamond.ac.uk/Beamlines/Materials/B16.html (accessed Oct 22, 2020).

(6) Sawhney, K. J. S.; Dolbnya, I. P.; Tiwari, M. K.; Alianelli, L.; Scott, S. M.; Preece, G. M.; Pedersen, U. K.; Walton, R. D. A Test Beamline on Diamond Light Source. In *AIP conference proceedings*; 2010; Vol. 1234, pp 387–390.

(7) Sawhney, K. J. S.; Dolbnya, I. P.; Scott, S. M.; Tiwari, M. K.; Preece, G. M.; Alcock, S. G.; Malandain, A. W. A Double Multilayer Monochromator for the B16 Test Beamline at the Diamond Light Source. In *Advances in X-Ray/EUV Optics and Components VI*; 2011; Vol. 8139, p 813908.

(8) Source, D. L. Diamond I15 Information http://www.diamond.ac.uk/Beamlines/Engineering-and-Environment/I15-Extreme.html (accessed Oct 22, 2020).

(9) Canberra. Canberra Partially Depleted Series Diode http://www.canberra.com/products/detectors/pips-detectors-standard.asp (accessed Oct 10, 2018).

(10) Rial, E. C. M.; Schouten, J. C. Electron Beam Heating Effects in Superconducting Wigglers at Diamond Light Source. *IPAC10* **2010**, 3195.

(11) Winick, H.; Brown, G.; Halbach, K.; Harris, J. Wiggler and Undulator Magnets. *Phys. Today* **1981**, *34* (5), 50–63.

(12) Cussler, E. *Diffusion: Mass Transfer in Fluid Systems*; Cambridge university press, 2009.

(13) Crank, J. *The Mathematics of Diffusion*; Oxford university press, 1979.

(14) Pedregosa, F.; Varoquaux, G.; Gramfort, A.; Michel, V.; Thirion, B.; Grisel, O.; Blondel, M.; Prettenhofer, P.; Weiss, R.; Dubourg, V.; Vanderplas, J.; Passos, A.; Cournapeau, D.; Brucher, M.; Perrot, M.; Duchesnay, E. Scikit-Learn: Machine Learning in {P}ython. *J. Mach. Learn. Res.* **2011**, *12*, 2825–2830.

(15) Moré, J. J. The Levenberg-Marquardt Algorithm: Implementation and Theory. In *Numerical analysis*; Springer, 1978; pp 105–116.

(16) Levenberg, K. A Method for the Solution of Certain Non-Linear Problems in Least Squares. *Q. Appl. Math.* **1944**, *2* (2), 164–168.

(17) Marquardt, D. W. An Algorithm for Least-Squares Estimation of Nonlinear Parameters. *J. Soc. Ind. Appl. Math.* **1963**, *11* (2), 431–441.

(18) Sánchez del Río, M.; Dejus, R. J. XOP v2.4: Recent Developments of the x-Ray Optics Software Toolkit. In *Advances in Computational Methods for X-Ray Optics II*; Sanchez del Rio, M., Chubar, O., Eds.; SPIE, 2011; Vol. 8141, p 814115. https://doi.org/10.1117/12.893911.

(19) Hubbell, J.; Seltzer, S. M. *NIST: X-Ray Mass Attenuation Coefficients*; 1996.

(20) Gevantman, L. H. Solubility of Selected Gases in Water. *Nitric oxide (NO)* **2000**, *308* (3.348), 10–14.
